# Insects shape the cadaver decomposition microbiome and postmortem interval estimation accuracy

**DOI:** 10.1128/msystems.00681-25

**Published:** 2026-04-27

**Authors:** Victoria Nieciecki, Valerie A. Seitz, Zachary M. Burcham, Kristen Otto, Kalen Cantrell, Jonathan Kirkland, Gail Ackermann, Rob Knight, Aaron Lynne, Jessica L. Metcalf, Sibyl Bucheli

**Affiliations:** 1 Department of Animal Sciences, Colorado State University 728642, Fort Collins, Colorado, USA; 2 Graduate Program in Cell & Molecular Biology, Colorado State University 3447, Fort Collins, Colorado, USA; 3 Department of Computer Science and Engineering, University of California San Diego 214553, La Jolla, California, USA; 4 Bioinformatics and Systems Biology Program, University of California San Diego 8784, La Jolla, California, USA; 5 Department of Pediatrics, University of California San Diego 547075, La Jolla, California, USA; 6 Shu Chien-Gene Lay Department of Bioengineering, University of California San Diego 207027, La Jolla, California, USA; 7 Halıcıoğlu Data Science Institute, University of California San Diego 684628, La Jolla, California, USA; 8 Center for Microbiome Innovation, University of California San Diego 8784, La Jolla, California, USA; 9 Department of Biological Sciences, Sam Houston State University 171821, Huntsville, Texas, USA; University of Toronto, Toronto, Ontario, Canada

**Keywords:** decomposition, microbiome forensics, Calliphoridae, blow flies, maggots, larvae, indoors, built environment, *Ignatzschineria*, *Thiopseudomonas*

## Abstract

The breakdown and recycling of carrion is a crucial ecological process that largely relies on a community of necrophagous insects and microbes. Recent work has shown that a specialized microbial network, likely dispersed throughout the environment by insects, assembles during cadaver decomposition to break down flesh regardless of climate and geography. Because of their broad distribution and successional nature, decomposer microbes have been used in machine learning models to predict the postmortem interval (PMI) of human remains, an important contribution to the field of forensics. How factors such as an indoor environment, which alters insect activity, impact the composition of microbial communities decomposing human remains is unclear. Here, we investigate the effects of enclosed shelter on microbial community assembly and successional patterns during human decomposition and provide important considerations for PMI modeling. Compared to outdoor cadavers, we show that indoor cadavers experienced delayed colonization of key decomposer microbes over the course of decomposition due to restricted insect access. Consequently, machine learning models trained on outdoor cadavers frequently underestimated the PMI of cadavers decomposing indoors. Delayed colonization by blow fly maggots (Diptera: Calliphoridae) was correlated with higher PMI prediction errors, suggesting that insects are an important source of microbial decomposers that drive PMI model predictions. Incorporating microbial data from indoor cadavers and insect activity into PMI models significantly improved prediction capabilities for both indoor and outdoor decomposition environments. Ultimately, we demonstrate the important role insects play in the maintenance and distribution of microbes that help to recycle vertebrate remains.

**IMPORTANCE:**

Microbes are critical for the decomposition and recycling of organic matter. Recently, microbiome-based models have shown promising performance in estimating the postmortem interval (PMI). However, many deaths occur indoors, yet no studies have investigated the impact of enclosed shelter on the cadaver microbiome in a controlled setting. Here, cadavers were decomposed indoors, and we found that blow fly maggots serve as an important source of decomposer taxa that significantly alter the cadaver microbiome following infestation. Notably, PMI estimation models trained on outdoor data sets failed to accurately predict the PMI when insect colonization was delayed. We show that incorporating 16S rRNA amplicon data from cadavers decomposing indoors, along with environmental variables, significantly improves PMI estimates, suggesting a microbiome-based forensic tool may be feasible across decomposition environments. Importantly, this research demonstrates the critical ecological role insects play in the dispersal of specialized microbes that are involved in the breakdown and recycling of vertebrate remains.

## INTRODUCTION

Carrion is an important and understudied detritus type, representing an ephemeral and patchy, high-quality nutrient pool (1–5). Although a small fraction of global biomass compared to plant detritus (6), carrion is important ecologically because of its high nutrient content, which attracts a diverse, multi-trophic suite of scavengers, including vertebrates, insects, and microbes (7–12). Recent work has advanced our understanding of the ecology and assembly of microbial decomposer communities. By decomposing cadavers across multiple climates, Burcham et al. (10) revealed that a conserved network of microbial decomposers assembles during human decomposition regardless of varied host communities, geography, and season. Key members of this network, which included *Thiopseudomonas alkaliphila* (previously *Oblitimonas alkaliphila*), *Ignatzschineria* spp., *Wohlfahrtiimonas chitinclastica*, *Vagococcus lutrae, Acinetobacter rudis,* and members from *Bacteroides, Savagea, and Peptoniphilaceae,* exhibited high cross-feeding potential, suggesting they likely co-metabolized the ephemeral nutrient pool that permeated the soil. While the mechanisms remain unclear, this work also provided evidence that insects and other scavengers likely play a large role in the dispersal of decomposer networks in the environment.

The ecological principles governing animal decomposition are applicable across many fields and may be used to improve the disposal and reuse of meat processing waste, enhance efficiency of the human death care industry, and develop new forensic tools. Recently, robust machine learning models based on the consistent succession of microbes across decomposing cadavers and carrion have been developed to predict time since death, or the postmortem interval (PMI) (10, 13–20). Although microbiome-based PMI estimation models are promising forensic tools, they are exclusively based on outdoor decomposition scenarios. A large number of human remains are found indoors, yet indoor decomposition is rarely investigated outside of case studies, and there has been no controlled research to date that has investigated microbial community succession patterns under indoor conditions in humans (21, 22). Of the few studies that have been conducted, several have observed that blow fly (Diptera: Calliphoridae) colonization of indoor cadavers is delayed compared to outdoor cadavers, and this delay is associated with slower decomposition rates (21, 23–26). The effect of insect colonization and enclosed shelter on the cadaver microbiome and microbial succession is currently unclear and represents a large scientific knowledge gap. As insects are likely critical for the dispersal of the decomposer network (12, 27–30), we hypothesized that microbial community succession will be significantly altered indoors if insect colonization is delayed. Furthermore, we suspect that these differences will likely negatively impact the accuracy of microbial-based PMI estimation models.

**Fig 1:**
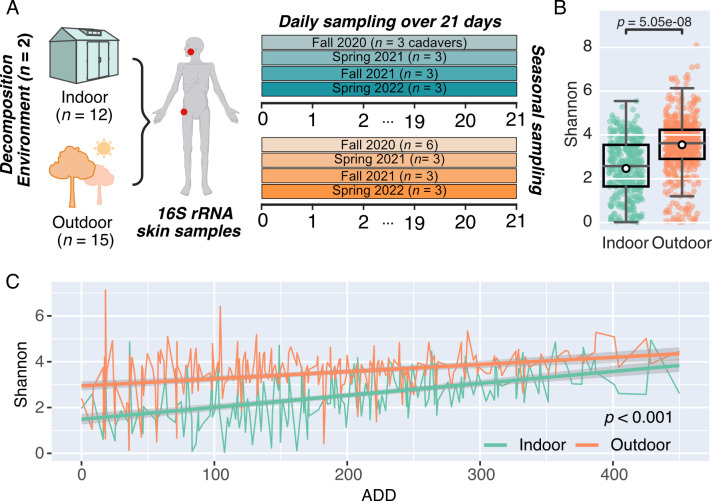
Alpha diversity differs between indoor and outdoor decomposition environments. (**A**) Experimental design—12 and 15 cadavers were decomposed inside enclosed shelters or outdoors, respectively. The skin of the face and hip was sampled daily for 16S rRNA amplicon sequencing (indicated by a red dot) for 21 days, beginning on the day of placement (day 0). Cadavers were placed in three indoor-outdoor pairs (except fall 2020; see figure) during the fall and spring over the course of 2 years. (**B**) Box plots comparing Shannon diversity of indoor (teal) and outdoor (orange) cadaver skin. The group median is represented by the centerline of the box plots. White markers represent the group mean values, adjusted for cadaver ID and ADD. A linear mixed-effects model was used to test for significance between groups. (**C**) Mean Shannon diversity is shown for indoor (teal) and outdoor (orange) cadaver skin samples across ADD, with standard error (gray). A linear mixed-effects model was used to test for statistical significance between indoor and outdoor environments across ADD, with cadaver ID set as a random intercept. Time points with multiple skin samples (face and hip) per cadaver were handled using nested random effects.

To better understand the impact of enclosed shelter on microbial community succession during human decomposition, over the span of 2 years during the spring and the fall, we placed a total of 12 cadavers inside unconditioned, enclosed shelters for human experiments on decomposition (SHEDs; also referred to as indoor[s] throughout this study) to naturally decompose, along with 15 outdoor control cadavers, at a forensic research facility in Huntsville, TX. Using 16S rRNA gene amplicon sequencing, we characterized cadaver skin microbial communities (skin of the face and hip) over the first 21 days of decomposition (Fig. 1A). We found that overall microbial community composition was strikingly similar between indoor and outdoor cadaver skin. However, indoor community succession lagged behind patterns observed in outdoor cadavers. Random forest models that incorporated microbiome data from indoor cadavers and environmental variables, such as evidence of maggots and accumulated degree day (ADD), a measure of accumulated temperature exposure over time, produced the most accurate PMI estimates, overcoming the different successional patterns observed during indoor decomposition. This novel research provides valuable insights about decomposer community assembly and demonstrates that machine learning model performance is closely tied to insect-cadaver interactions, a critical discovery that will advance the development of PMI prediction models.

## RESULTS

### Community differences between indoor and outdoor cadaver decomposition

Cadavers decomposing in an outdoor, aboveground environment are exposed to numerous environmental and biotic variables such as precipitation, insects, and soil that may be limited in indoor decomposition scenarios. We first sought to understand how enclosed shelter impacts the microbial communities that assemble on decomposing cadaver skin. On average, microbial communities taken from the skin of indoor cadavers had lower Faith’s phylogenetic diversity, Shannon diversity, and unique amplicon sequence variants (ASVs) compared to outdoor cadaver skin (Fig. 1B; Fig. S1A and B). We found that 7,592 ASVs were unique to outdoor cadavers, but this large number only accounted for an average relative abundance of 2.5% (Fig. S1C and D), suggesting that although less diverse on average, indoor skin communities share a large number of highly abundant ASVs with outdoor cadaver skin. While initially lower indoors (LME model *P*-value <0.001), Shannon diversity tended to increase with ADD and was similar to outdoor skin communities by the final day of decomposition (Fig. 1C).

**Fig 2:**
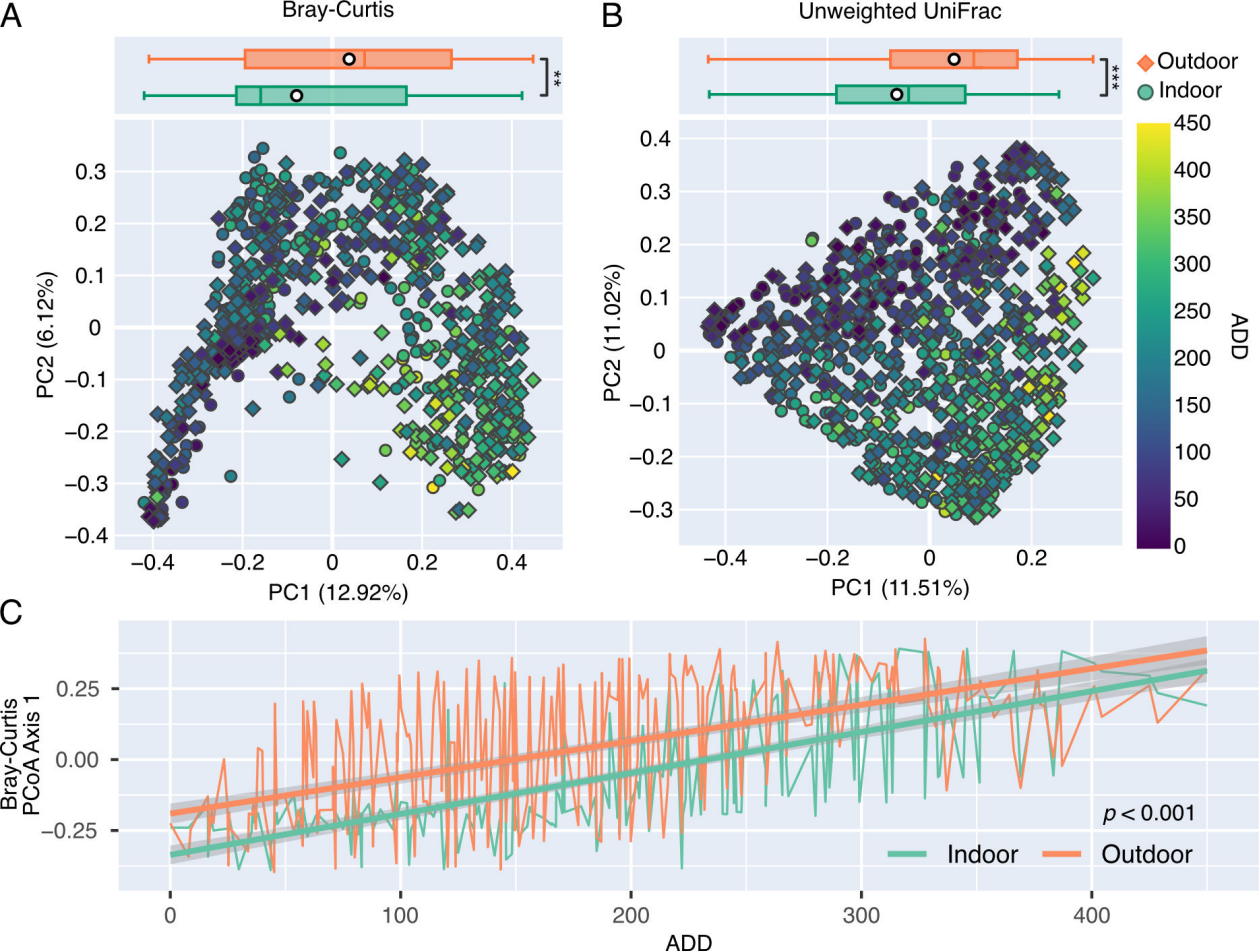
Indoor microbial communities differ from outdoor skin communities across decomposition. (**A**) Bray-Curtis and (**B**) unweighted UniFrac PCoA plots. Skin samples collected from outdoor (diamond) and indoor (circle) cadavers are colored by ADD. Box plots show the distribution of PCoA axis 1 coordinates for indoor (teal) and outdoor (orange) skin samples. White markers represent group means adjusted for cadaver ID and ADD. Linear mixed-effects models were used to test for significance between groups. **P-value < 0.01 and ***P-value < 0.001. (**C**) Mean Bray-Curtis PCoA axis 1 coordinates are shown for indoor (teal) and outdoor (orange) cadaver skin samples across ADD, with standard error (gray). A linear mixed-effects model was used to test for statistical significance between indoor and outdoor, with cadaver ID used as a random intercept. Time points with multiple skin samples (face and hip) per cadaver were handled using nested random effects.

Both Bray-Curtis and unweighted UniFrac beta diversity metrics showed that indoor and outdoor cadaver skin communities differed, and these differences were also significantly dependent on ADD (Fig. 2A and B). Bray-Curtis distances, and to a lesser extent unweighted UniFrac, showed greater variability between indoor and outdoor communities during early and active decomposition (0–200 ADD) along principal coordinate analysis (PCoA) axis 1 (Fig. 2C; Fig. S1E). Although statistically different across ADD (LME model *P*-value <0.001), indoor and outdoor microbial communities appeared to converge on similar compositions during advanced decomposition (>200 ADD), suggesting that other factors may play a larger role in shaping community structure, especially during advanced decomposition.

**Fig 3:**
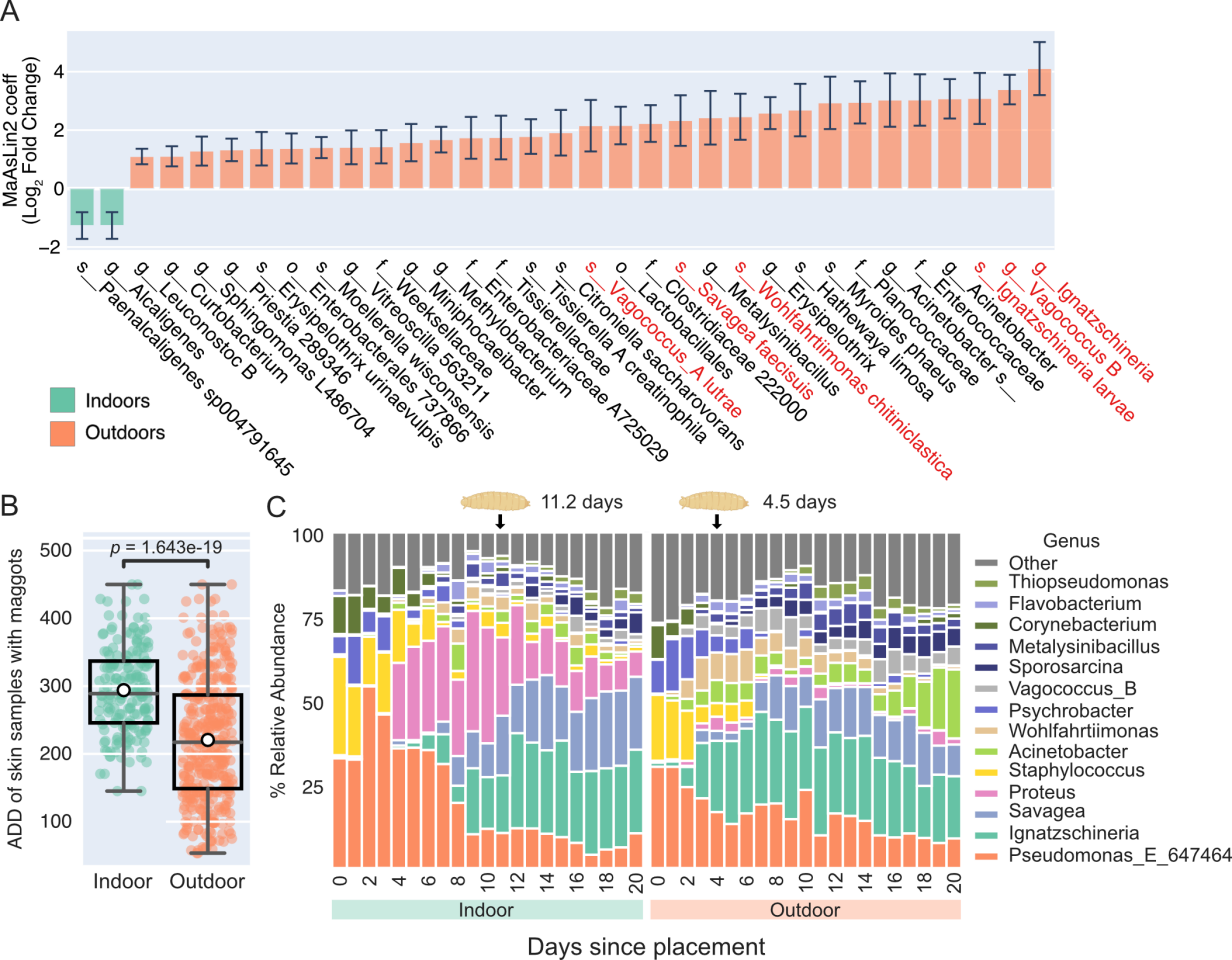
Maggot emergence is delayed by enclosed shelters. (**A**) Bar plot showing MaAsLin2 coefficients (log_2_ fold change) with standard error bars. ASVs were collapsed at the species level and are shown at the lowest taxonomic classification. Orange taxa were enriched in outdoor cadaver skin samples, while taxa in teal were enriched indoors. Taxa highlighted in red are common cadaver decomposers that likely originate from insects (10). Only taxa with ≥±1 log_2_ fold change and *q*-values <0.05 are shown. (**B**) Box plot shows the ADD of skin samples at the time of collection with evidence of maggots for indoor (teal) and outdoor (orange) cadavers. The group median value is represented by the centerline of the box plots. White circle markers show group mean values. Mann-Whitney *U*-test was used to test for significance between groups. (**C**) Taxa bar plots show average percent relative abundance of the 14 most abundant genera found in indoor (left) and outdoor (right) cadaver skin samples across all 21 days of decomposition. Maggot icons denote the average first day of notable maggot mass for each group.

We next evaluated which microbial taxa were differentially abundant between the skin of cadavers decomposing indoors and outdoors. The relative abundance of numerous microbes was enriched in the outdoor cadaver group, while only a few were more abundant on indoor cadaver skin (Fig. 3A). Importantly, several of the taxa enriched outdoors, including *Ignatzschineria larvae, Savagea,* and *Wohlfahrtiimonas chitiniclastica,* are key members of the universal decomposer network identified in previous research (10) and are strongly associated with blow flies, blow fly maggots, and beetles (12, 31). Enrichment of these taxa on outdoor cadaver skin, but not indoor, strongly suggests that insect activity may be an important driver of cadaver community assembly and composition.

### Influence of blow fly maggots on the decomposer microbiome

Decomposing vertebrates represent an important nutrient source that, immediately following death, is utilized by female blow flies to lay eggs that then emerge as larvae (maggots) and feed on the remains (32, 33). Blow fly access to and from the indoor structures was not controlled during this study, and consequently, the first appearance of maggots on indoor cadavers lagged behind their appearance on the cadavers placed outdoors. This difference was likely due to obstructed access into the SHEDs, which were only opened once per day for sampling. In support of this, the average first day of a notable maggot presence was 11.2 days (ADD = 203.7) after the placement of indoor cadavers compared to 4.5 days (ADD = 73) for outdoor cadavers (Fig. 3B). We observed that two indoor cadavers (IDs D7 and D9) and two outdoor cadavers (IDs D11 and D12) were not colonized by noticeable maggots during the timeframe of our study (21 days), which may have been due to low outdoor temperatures (mean outdoor temperature during decomposition = 11.7°C).

We found that cadaver skin without previous exposure to maggots was enriched for bacteria from the genera *Pseudomonas, Staphylococcus,* and *Corynebacterium* (Fig. S2). However, following their emergence, taxa known to be associated with maggots, such as *Ignatzschineria, Savagea, Vagococcus, Thiopseudomonas alkaliphila,* and many others, significantly increased in relative abundance. Taxa bar plots that were grouped by decomposition environment and ordered by days post placement show that the relative abundance of these maggot-associated taxa begins to increase concurrently with maggot emergence (Fig. 3C; Fig. S3 and S4). This aligns with the Bray-Curtis PCoA plot where indoor and outdoor communities became more similar to each other by advanced decomposition (~200 ADD). Interestingly, cadavers in which maggots were never detected were almost entirely dominated by *Pseudomonas, Staphylococcus,* and *Corynebacterium* across all 21 days of decomposition (Fig. S3 and S4). Together, this evidence supports the idea that significant changes in microbial community composition are associated with notable maggot mass development.

**Fig 4:**
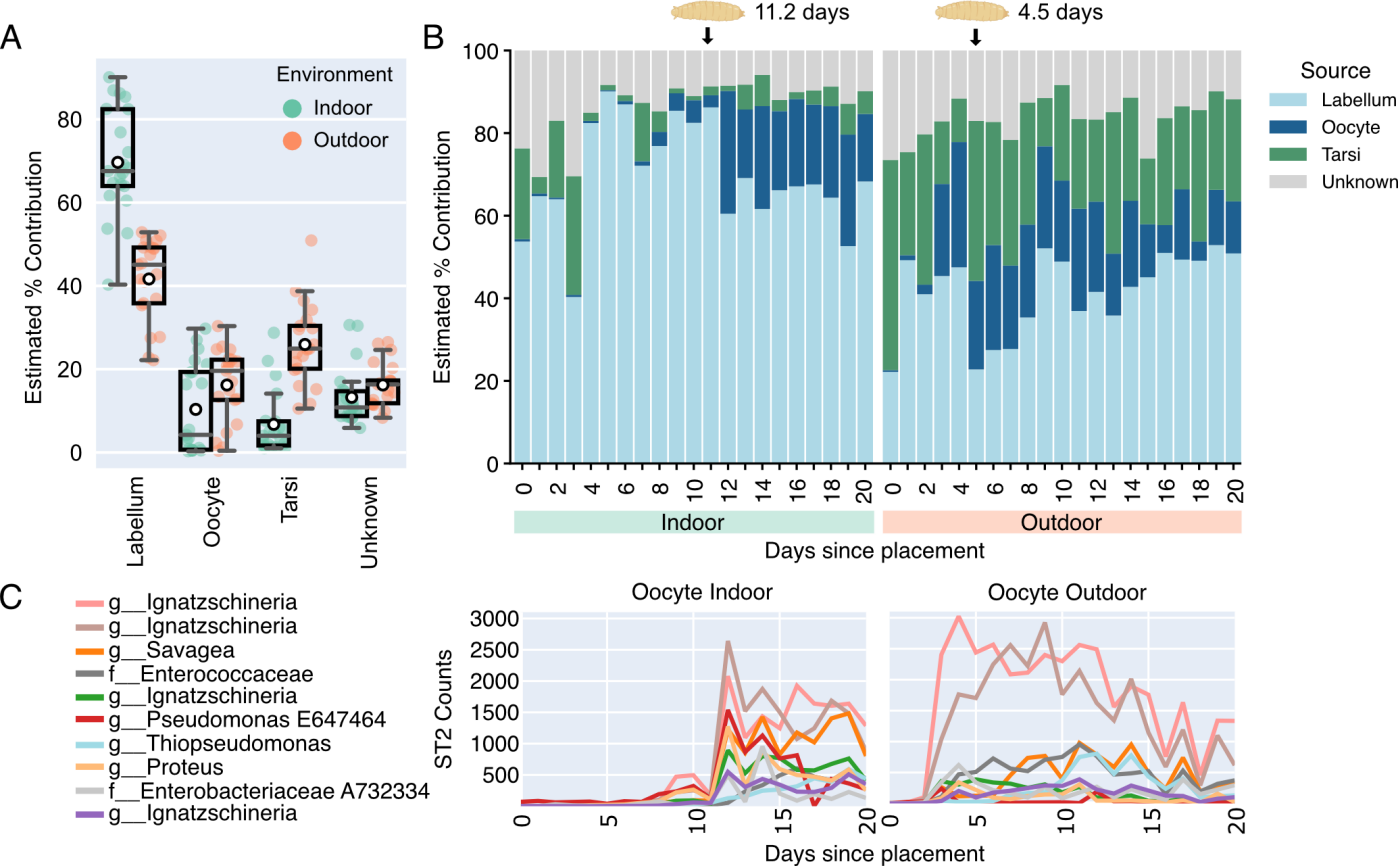
Microbial transfer between blow flies and cadaver skin. (**A**) SourceTracker2 (ST2) was used to estimate the amount each blow fly organ microbial community contributes to the cadaver skin microbiome. Box plots show the estimated percent contribution from each fly organ to indoor (teal) and outdoor (orange) cadaver skin. The group median value is represented by the centerline of the box plots. White markers represent the group mean value. (**B**) Estimated percent contribution from each blow fly organ (labellum, light blue; oocyte, dark blue; tarsi, green) to indoor (left) and outdoor (right) cadaver skin across 21 days of decomposition. Unknown (gray) represents the proportion of sequences that may have originated from an unknown source. (**C**) Feature assignment counts (ST2 Counts) for the 10 ASVs with the highest contribution assigned to oocytes are plotted across days of decomposition for indoor (left) and outdoor (right) cadaver skin. Taxa are labeled at their lowest taxonomic assignment.

We next used microbial source tracking to better understand the transfer of microbes between different blow fly organs (labellum, tarsi, and oocytes) (31) and cadavers over time. Across both environments, blow fly labellum contributed the largest proportion of microbial communities to cadaver skin (indoor mean = 69.6%; outdoor mean = 41.7%; Fig. 4A). In outdoor cadavers, tarsi contributed the second largest proportion of communities (mean = 25.9%), whereas in indoor cadavers, blow fly oocyte contributed the second highest proportion (mean = 10.3%). Within both environments, the contribution of microbial communities from blow fly oocytes increased around the same time the first signs of a maggot infestation were evident (Fig. 4B). The predominant contributions from labellum and tarsi on the day of placement prior to blow fly activity and during early decomposition were largely driven by *Pseudomonas* and *Staphylococcus* ASVs (Fig. S5), which is consistent with the transfer of cadaver skin microbes to blow fly organs during initial contact. Meanwhile, we found that the contribution from oocytes was primarily driven by two *Ignatzschineria* ASVs and one *Savagea* ASV (Fig. 4C), suggesting that blow fly eggs have a distinct microbiome that is transferred to cadavers via maggot infestation and feeding.

### Predicting the indoor postmortem interval using a model trained on outdoor cadavers

**Fig 5:**
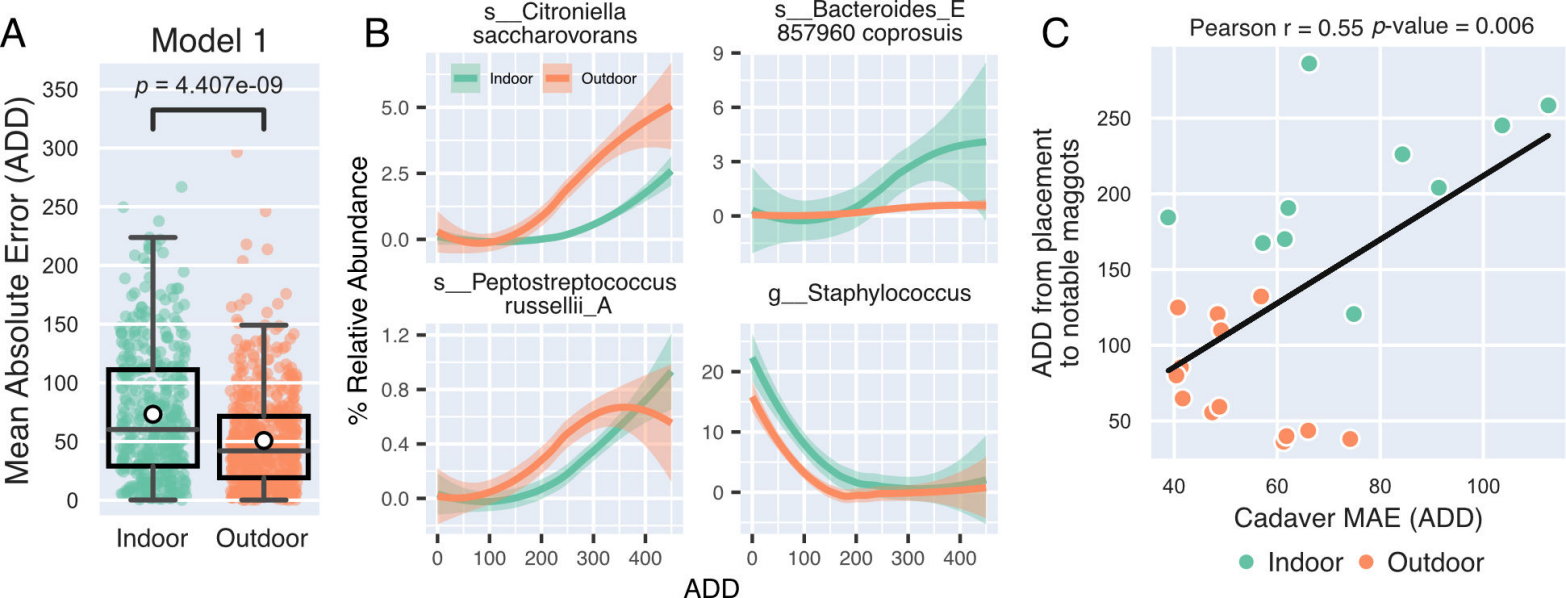
Maggots drive outdoor-based PMI model predictions. (**A**) RFR model trained on skin samples collected from cadavers decomposing at three different outdoor forensic research facilities was used to predict the PMI of a new indoor and outdoor cadaver data set. Box plot of MAE of prediction is shown for both groups. White marker denotes group means. Mann-Whitney *U*-test was used to test for statistical significance. (**B**) The four most important Model 1 features are shown from most (top left) to fourth most important (bottom right). The percent relative abundance of each feature is compared between indoor (teal) and outdoor (orange) cadaver skin samples across decomposition. Percent relative abundances were smoothed using LOESS regression. (**C**) Scatter plot shows MAE of each cadaver plotted against the time (ADD) between cadaver placement and the first appearance of maggots. Cadavers that were never colonized by maggots were omitted. Colors represent decomposition environments. Pearson correlation (*r*) was used to test for significant correlations between variables. Linear regression of MAE and maggot ADD is shown in black.

Although indoor microbial succession patterns appeared to vary from outdoor cadavers, both cadaver groups shared similar taxa, which may enable the use of the large and robust Burcham et al. (10) data set (*n* = 36 outdoor cadavers) for predicting PMI regardless of decomposition environment (indoor vs outdoor). We found that the Burcham et al. (10) data set and the data set generated as a part of this study shared 2,153 model features, which represent ASVs grouped at the species level. The random forest regression (RFR) model (Model 1) trained on microbial data collected from the cadavers decomposing outdoors performed better with the outdoor cadavers in this study. The mean absolute error (MAE) was 50.86 ADD for outdoor cadavers (*n* = 15) compared to 73.26 ADD for indoor cadavers (*n* = 12; Fig. 5A). We also found that this model generally underpredicted the ADD of indoor cadavers, especially at later time points (Fig. S6A).

We next looked to identify potential differences between indoor and outdoor cadaver communities from this study that could be driving the significant differences in prediction accuracy by examining the 20 most important taxa to Model 1 (Fig. S6B). The change in relative abundance of each taxon across ADD was used to examine possible differences in taxa arrival time or in magnitude. Many of these important features exhibited similar trends (e.g., relative abundance increased across ADDs), but these changes were generally delayed in the indoor group. For example, the most important Model 1 feature, *Citroniella saccharovorans*, began to increase at approximately 150 ADD in outdoor cadavers and at approximately 275 ADD in indoor samples (Fig. 5B). Similarly, *Peptostreptococcus russelilli_A* increased at approximately 100 ADD in outdoor samples, while in indoor samples, this taxon was delayed until 200 ADD (Fig. 5B).

Of the 20 most important taxa from Model 1, 13 were also significantly enriched in cadaver skin samples with notable maggots (Fig. S2) suggesting a possible link between model performance and maggot-associated taxa. We also found that many of the important features were generally absent from the cadavers that were never colonized by maggots (Fig. S7). Based on the likely association between these important model features and maggots, we evaluated whether PMI prediction MAEs and ADD at the time of first notable maggot appearance were correlated. For most cadavers, PMI errors increased as the time between cadaver placement and first maggot appearance increased (Fig. 5C). This trend strongly indicates that blow fly- and maggot-associated microbes drive PMI modeling.

### Incorporating indoor cadavers and environmental variables into PMI models

**Fig 6:**
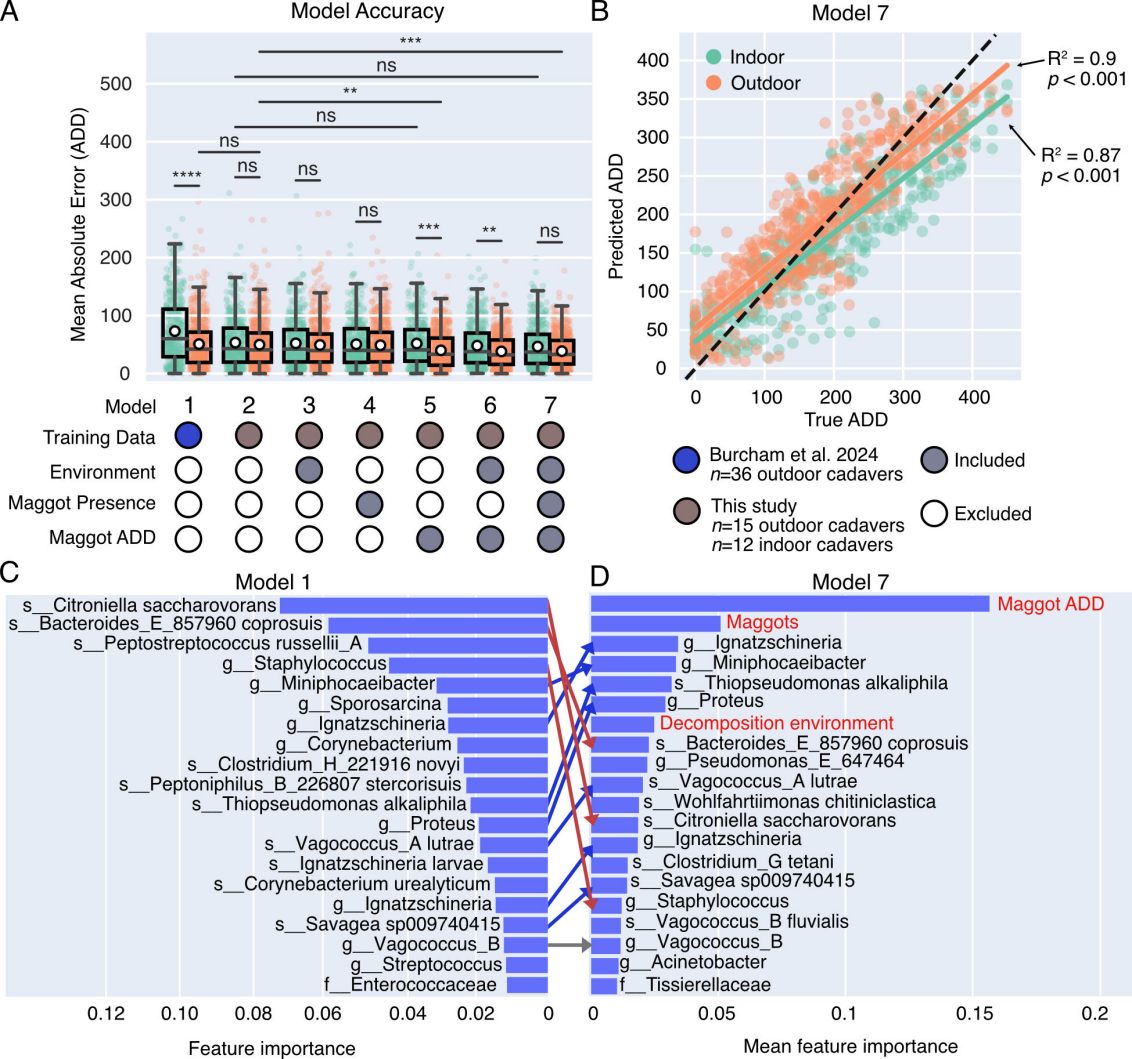
Indoor cadaver data and environmental variables improve model accuracy. (**A**) Box plots show mean absolute errors (in ADD) for each model. Sample predictions are grouped according to indoor (teal) and outdoor (orange) cadavers. White markers represent group means. The table summarizes which data types were included in each model. Training data denotes the 16S rRNA amplicon data set used to train each model. Accuracy for all models was evaluated using the current study as the test set. Open or closed gray circles denote whether each variable (decomposition environment, maggot exposure, and maggot ADD) was excluded or included in each model, respectively. ANOVA with *post hoc* Tukey HSD correction for multiple comparisons was used to test for significance: ****P-value < 0.0001; ***P-value < 0.001; **P-value < 0.01; ns, not significant. (**B**) Scatter plot displaying ADDs predicted by Model 7 compared to true ADDs for indoor (teal) and outdoor (orange) skin samples. Linear regressions for indoor and outdoor groups are shown as solid lines. The dashed black line represents perfect prediction accuracy. (**C**) The 20 most important Model 1 features are displayed from most important (top) to least important (bottom). Taxa were labeled using the lowest taxonomic classification. (**D**) The 20 most important features from Model 7 are shown from most important (top) to least important (bottom). Taxa were labeled using the lowest taxonomic classification. Environmental variables are colored in red. The average of each feature was taken across all cross-validation folds. Arrows connecting features in panels C and D highlight the changing order of important taxa between the two models. Features with no corresponding line were unique to the top 20 features of that model. Red arrows highlight taxa that dropped in importance, while blue arrows show taxa that increased between models. Taxa with gray arrows did not change.

Given that most outdoor cadavers are almost immediately visited by blow flies, RFR models trained solely on outdoor data likely fail to capture microbial succession patterns that arise when maggot colonization is delayed. To account for this, we next trained and tested several RFR models on the data collected during this study to determine if including indoor cadavers and other environmental variables could help improve PMI prediction accuracy for indoor cadavers. In total, six additional models were trained (Models 2–7), which incorporated information about the decomposition environment (whether the cadaver was inside or outside), maggot exposure (whether there was a maggot mass at the time of sampling), and the ADD of the maggots at the time of sampling, alone or in combination (see Fig. 6A).

Combining microbial data from indoor and outdoor cadavers into one model (Model 2) significantly improved indoor prediction errors from 73.3 ADD to 52.2 ADD (*P*-value *<* 0.0001; Fig. 6A). Importantly, the addition of indoor cadavers had no significant effect on outdoor prediction performance (Model 2; 50.9 ADD vs 49.3 ADD; *P*-value > 0.05). Including decomposition environment or previous maggot exposure data (Models 3–4) did not further improve overall (combined indoor/outdoor) predictions (Fig. S8). However, when the ADD of the maggots at the time of sample collection was also included (Fig. 6A; Model 5), PMI MAE of outdoor, but not indoor, samples significantly decreased (*P*-value = 0.0028). Overall, the model that included all data types (Fig. 6A; Model 7) yielded the highest prediction accuracy across both decomposition environments (indoor MAE = 46.6 ADD; outdoor MAE = 38.6 ADD) despite the variation in maggot emergence times (Fig. 6A and B).

We found the most important microbial features underpinning Model 1 (Fig. 6C), which was only trained on outdoor cadavers, were similar to those driving the best-performing model (Model 7; Fig. 6D). However, the order of importance was altered—*Ignatzschineria, Miniphocaeibacter*, *Thiopseudomonas alkaliphila*, and *Proteus* became the most important microbial features following maggot variables. To better understand whether these important features were dependent on the presence of maggots, we examined their relative abundance in maggot-free cadavers over ADD and found three of the top four most important Model 1 features were entirely absent or almost undetectable in maggot-free cadavers (Fig. S7). In comparison, only *Miniphocaeibacter* was absent, while *Ignatzschineria, Thiopseudomonas alkaliphila,* and *Proteus* were found in low to medium abundance in maggot-free cadavers (Fig. S9A) suggesting this new model relies less on maggot-associated features, which likely enables accurate predictions across varying degrees of insect activity. In total, we found that 8 of the 17 most important microbial features to the best-performing model (Model 7) were entirely absent or almost undetectable from maggot-free cadavers. Even so, the maggot-free cadavers still predicted well (Fig. S9B), demonstrating the robustness of this PMI prediction model against missing features.

## DISCUSSION

Our results demonstrate that cadaver skin microbial succession during decomposition is altered by enclosed structures due to delayed colonization by blow flies and their associated microbes. This finding is similar to other indoor decomposition studies that also reported delayed necrophagous insect colonization (but did not study their microbes) (21, 23, 24, 26). In agreement with Burcham et al. (10), we found necrophagous insects, particularly blow flies, appear to be the main dispersal mechanism of most key microbes involved in decomposing animal flesh, which in most outdoor scenarios occurs almost immediately upon death. Microbial source tracking revealed that some *Ignatzschineria* ASVs important for PMI modeling likely originate from blow fly oocytes that develop into maggots 24–48 hours following oviposition (34). We observed that on outdoor cadavers, oocyte-associated *Ignatzschineria* ASVs increase 1.5 days before notable maggot masses appear, while on indoor cadavers, these ASVs increase shortly after maggot masses appear (Fig. 4C). We suspect this discrepancy was due to higher outdoor blow fly activity and oviposition, thereby increasing the probability of sampling *Ignatzschineria* ASVs. Conversely, indoors, where there were fewer blow flies, *Ignatzschineria* was likely spread from eggs to cadaver skin through maggot migration and feeding activities. However, egg observations, including oviposition times, were not recorded during this study. Additionally, the blow fly maggot microbiome was not characterized, making it difficult to assess the respective contributions of blow fly eggs and maggots to the cadaver skin microbiome and will require further research.

We confirm here that delayed blow fly maggot colonization of indoor cadavers has large implications for microbial-based PMI estimation models. However, we find that PMI prediction errors can be overcome by incorporating microbial community data from indoor cadavers into PMI models and several additional environmental variables, including whether the cadaver was decomposing inside or outside, whether there was evidence of maggot colonization, as well as the ADD of the maggots at the time of sampling (Fig. 6; Fig. S6). Even so, we observed that prediction accuracy begins to decrease during advanced decomposition (>200 ADD) regardless of decomposition environment (Fig. 6B). These higher errors are likely due to several factors. Fewer cadavers reach higher ADD values within the 21 day decomposition period, which results in fewer model training points, but this tends to only impact ADD values greater than 400. More importantly, we observed the relative abundance of several taxa that are important for modeling (e.g., *Peptostreptococcus russellii*) increased during early decomposition, then decreased during late-stage decomposition (Fig. 5B). This relative decrease in abundance likely occurs concurrently with a shift in nutrient pools from readily available carbohydrates and proteins to less labile lipids (10, 35, 36). For instance, the relative abundance of *Corynebacterium* increased during advanced decomposition (Fig. S7). Given that members of this genus are known to metabolize lipids (e.g., *Corynebacterium urealyticum*) (37), we hypothesize the observed decline in prediction accuracy likely reflects the fluctuation of available nutrients and microbial community members that can metabolize these nutrient pools. Characterizing these late-stage microbial processes will be essential for improving PMI estimates during advanced decomposition.

Although several indoor and outdoor cadavers were never colonized by blow fly maggots, PMI was still accurately predicted for these individuals. Burcham et al. (10) demonstrated that the most important decomposers for PMI estimation exhibited similar trends across decomposition locations and across multiple climates, enabling a generalizable PMI model. Here, we show that even when some of the most important insect-derived features are missing from a subset of samples (i.e., maggot-free cadavers; Fig. S9), RFR models are still able to accurately predict PMI by leveraging other taxa. For example, the maggot-free cadavers exhibited a meat spoilage-like microbiome which is often characterized by a low-diversity microbial community dominated by *Pseudomonas* (38–40). In a study investigating chicken carcass chilling methods, Belk et al. (40) noted that water chilling decreased bacterial load and hypothesized that this depleted biomass, in turn, reduced microbial competition which led to *Pseudomonas* overgrowth. We suspect a similar ecological process occurs during human decomposition. If maggot-associated microbes fail to inoculate human remains, *Pseudomonas* or species that are likely native to the cadaver may come to dominate the ecosystem. Indeed, we observed that *Pseudomonas, Staphylococcus*, or *Acinetobacter* dominated most indoor and outdoor maggot-free cadavers over time, a trend which likely drives PMI estimates of these cadavers and enables an insect-independent PMI model. Therefore, we anticipate our model would perform well under various blow fly colonization scenarios, including immediate, delayed, and no colonization. We hypothesize this type of model may even improve the prediction of cadavers decomposing during colder months when insect activity is low or absent.

Consolidating multiple large decomposition data sets into a single model that can accurately estimate PMI across various geographical locations, seasons, decomposition environments, and insect activity levels within a forensically useful timeframe is a large challenge. Whether one large, mixed decomposition scenario model is feasible or whether multiple smaller, scenario-specific models are needed to optimize model performance is currently unclear, and additional research is needed to inform best practices. In this study, the best-performing model incorporated 0.8 indoor cadavers for each outdoor cadaver, a balance that maintained outdoor prediction performance while enabling accurate indoor predictions. The results presented here, along with those demonstrated by Burcham et al. (10), suggest that an expanded model with a mixture of microbial and insect data from various indoor and outdoor geographical locations may be possible to construct. However, whether maintaining a near 1:1 indoor-to-outdoor cadaver ratio will be needed to maintain performance is currently unclear. Furthermore, the enclosed structures used in this study were unconditioned and closely followed daily outdoor temperature fluctuations (Fig. S10). We expect that indoor decomposition patterns may be further altered in conditioned spaces with stable temperatures. Microbial enzymatic activity tends to be more efficient at steady temperatures (41), which may result in accelerated cadaver decomposition compared to cadavers decomposing under similar but fluctuating temperatures. Consequently, our current model may overestimate ADDs in these scenarios, but further research is needed.

Our results also bring up several interesting questions about the relationship between necrophagous insects and their associated microbes. For example, do these microbes provide fitness advantages to the insects that host them? Are some key decomposers only found on particular species of insects such as blow flies or carrion beetles (adults versus larvae, females versus males)? Furthermore, do insect-associated microbial communities from different insect species compete for decomposition resources or do they cooperatively break down the nutrient pool? Future research is needed to unravel these relationships and answer these questions, which could additionally help refine PMI models as our understanding of the interdependency between insects and decomposer microbes increases.

## MATERIALS AND METHODS

### Study design, cadavers, and cadaver placement

We utilized the Sam Houston State University Southeast Texas Forensics Facility (STAFS) to place willed-body cadavers in indoor and outdoor decomposition scenarios. STAFS is a morgue-like laboratory located approximately 5 km north of Huntsville, TX, with an outdoor area designed for decomposition studies and forensic science training. The facility is located in the southeast Texas Pineywoods ecoregion, characterized by a humid and subtropical climate and a sparse forest covering of pine trees and a ground covering of herbaceous plants. The soil is a fine, friable sand that is moderately to strongly acidic and is moderately well-drained, with medium available water content and slow permeability and runoff (42).

Cadavers did not undergo autopsy, nor were they embalmed. All cadavers were stored at 4°C until placement. Individuals who died from SARS-CoV-2 or related complications were excluded from the study.

We placed a total of 27 human cadavers to decompose naturally between the fall of 2020 and the spring of 2022 (indoors *n* = 12; outdoors *n* = 15). During the fall of 2020, three cadavers were placed inside individual enclosed SHEDs, along with six outdoor cadavers (*n* = 3 caged, *n* = 3 uncaged). During the spring and fall of 2021 and the spring of 2022, three cadavers were placed indoors with three paired outdoor cadavers per season, for a total of nine indoor and nine outdoor (all caged) cadavers. Cadavers were placed in indoor-outdoor pairs approximately one week apart for three consecutive weeks during each placement season.

The enclosed SHEDs were constructed from plastic (Rubbermaid 7-ft × 7-ft Resin Outdoor Storage Shed) and were not temperature controlled. Cadavers were placed directly on the plastic floor and left to naturally decompose. Doors remained closed throughout the duration of the study unless sampling was taking place. Outdoor cadavers (*n* = 15) were placed on undisturbed ground. All cadavers were placed unclothed in a supine position. A scavenger exclusion cage, constructed from wood and wire, was placed over 12 of the 15 outdoor cadavers to prevent loss of limbs and other body parts to scavengers, as has been done in previous research at this site (10, 13). Scavenger exclusion cages were not utilized for three outdoor cadavers. The three uncaged outdoor cadavers were placed with the indoor-outdoor pairs during the fall of 2020.

### Temperature measurements and accumulated degree day calculations

Daily average air temperature was collected from the Easterwood Airport Station using Weather Underground (https://www.wunderground.com/). Temperature loggers were placed inside each of the three SHEDs (Onset HOBO MX2201 Pendant Wireless Temperature Data Logger). Indoor temperature was logged every 61 minutes, and the average was taken for each day. Temperature loggers collected indoor temperatures from 2 December 2020 until 13 May 2021, at which time all three loggers experienced a malfunction. The temperature of all three SHEDs was compared against the average outdoor temperatures during this period to determine whether outdoor temperatures could be substituted to calculate ADD. We found the indoor temperatures were highly correlated with outdoor temperatures (Pearson’s *r* = 0.96, *P*-value < 0.001; Fig. S10B) and varied on average by ±1.4°C. We therefore used daily average outdoor temperature to calculate ADD for both indoor and outdoor cadavers.

ADD estimates were calculated following Megyesi et al. (43). On day 0, the day of placement, ADD was set to zero for each cadaver. The ADD of the following day (day 1) was calculated as the average temperature of the preceding day (day 0). On day 2, the ADDs of all preceding days (day 0 + day 1) were summed. This was repeated for all succeeding days so that the final day’s ADD (day 21) was the sum of all the preceding average daily temperatures. Values below 0°C (baseline) were excluded.

Maggot ADD was calculated as described above; however, maggot ADD calculations began on the first day maggots or a maggot mass was observed. Maggots were considered present if 20 or more were observed on the cadaver at the time of sampling or a mass (cluster) of 20 or more maggots was present. Additionally, we only considered maggots that were present on the cadaver for at least 2 consecutive days. Once maggots were established on a cadaver, ADDs were calculated from the first day of maggot appearance until the final day of decomposition (21 days after cadaver placement). That is, maggot ADDs were not reset if a secondary colonization event occurred. We used the rationale that once maggots were established, maggot-to-cadaver microbial transfer has likely occurred. Additionally, forensic entomologists frequently use blow fly development stage and pupa casings to estimate cadaver ADD, making maggot ADD a forensically useful metric.

### Sample collection

Samples were collected using sterile dual-tipped BD SWUBE Applicator (REF 281130) swabs. For the first 21 days of decomposition, samples were taken daily, beginning on the day of placement (day 0), one from the skin of the face and another from the hip. Skin samples were collected by gently rubbing the swab over the skin area for approximately 30 seconds, with care not to break or damage the skin. For outdoor cadavers, the soil outside of the purge zone near the hip was also sampled with a swab until visible soil material adhered to the swab. Skin swabs were immediately transferred to a −20°C freezer at Sam Houston State University until they were shipped on dry ice to the Metcalf lab at Colorado State University for DNA extraction and amplicon data generation.

### DNA extraction

Each swab from the dual-tipped SWUBE was trimmed using sterile scissors and placed into a barcoded 96-well plate. One swab head was used for DNA extractions, and the other was stored at −20°C as a backup in the respective 96-well plate. The MagAttract PowerSoil Pro DNA isolation kit (Qiagen, cat no. 47109) for KingFisher was used to extract microbial DNA following the manufacturer’s instructions with the following modifications. DNA extraction plates 1 and 2 were processed using the 96-well PowerBead Pro plate (Qiagen). All remaining DNA extraction plates were processed using a single bead tube format in place of a 96-well PowerBead Pro plate until lysate was transferred to a KingFisher Flex System (Thermo Fisher). Following lysis and inhibitor removal, we utilized all lysate using a two-step binding approach outlined in the Earth Microbiome Project (EMP) DNA extraction protocol (44). Briefly, all lysate was split between two deep-well KingFisher plates containing equal parts of binding solution (QSB1). MagAttract Suspension G beads were added to the DNA binding plate 1 before transferring to a KingFisher for binding, purification, and elution. DNA was eluted in 60 μL of solution C6. Each DNA extraction plate included six blank extraction controls where no sample was added and two positive DNA controls where 25 μL of ZymoBIOMICS Microbial Community Standard was added prior to extraction. Samples were randomized across all plates to reduce batch effects.

### Amplicon library preparation and sequencing

The V4 region of the 16S rRNA gene was amplified following the EMP protocol (45) using Golay barcoded primers 515F (5′GTGYCAGCMGCCGCGGTAA) (46) and 806R (5′GGACTACNVGGGTWTCTAAT) (47). All samples were amplified to 30 cycles using the Platinum Taq DNA Polymerase Kit (Invitrogen), with the exception of DNA extraction plates 14–18, which were amplified to 27 cycles due to *Achromobacter* contamination originating from the MagAttract Suspension G beads (see below). Following amplification, 16S amplicons were quantified using the Quant-iT PicoGreen dsDNA Assay Kit (Invitrogen). Each PCR plate was pooled together in equimolar amounts and cleaned using UltraClean PCR Clean-Up Kit (Qiagen). Libraries were shipped on ice to the CSU Foothills Campus sequencing core or to the Fierer lab at the University of Colorado, Boulder, for sequencing. 16S rRNA amplicon libraries were sequenced using a 500-cycle kit on the Illumina MiSeq platform.

### Achromobacter contamination

Contamination was discovered in the negative extraction controls on DNA extraction plates 14–18 during PCR amplification in October 2022. Each reagent in the MagAttract PowerSoil Pro DNA isolation kit (Qiagen, cat no. 47109) for KingFisher was tested individually to determine the source. This was done by combining each potentially contaminated reagent with 600 μL of CD3 bind from a clean DNeasy PowerSoil Pro spin column kit (Qiagen cat. no. 47014) and proceeding with the remaining steps in the DNeasy PowerSoil Pro spin column kit protocol with several exceptions. To test CD1, 800 μL was added to a clean bead tube and vortexed on high for 10 minutes. The lysate was combined with 300 μL of clean CD2, centrifuged, and the supernatant was then mixed with clean CD3 for binding and cleanup. CD2 was tested by combining with 800 μL of clean CD1. The solution was pelleted, and then clean CD3 was added to the supernatant. Thirty microliters of MagAttract Suspension G beads was tested by eluting in 450 μL of clean C6. This elution was then combined with 600 μL of clean CD3 bind. PCR was then done as described above. Positive amplification was observed in the MagAttract Suspension G beads (lot #172029496; Fig. S11A).

To determine if contamination from the MagAttract Suspension G beads could be computationally removed from DNA extraction plates 14–18, we re-extracted a subset of 13 samples using a clean MagAttract PowerSoil Pro DNA isolation kit (Qiagen, cat no. 47109) for comparison. Contaminated samples were re-amplified to 27 cycles, which produced clean negative extraction controls compared to those amplified to the standard 30 cycles (Fig. S11B). The re-extracted subset of clean samples was amplified to the standard 30 cycles. Libraries were prepared for sequencing as described above. Raw sequencing data were processed as described below. The V4 99% Silva 138 naïve Bayes classifier was used to classify taxonomy. We found that the contamination largely originated from a single *Achromobacter* sequence (5′TACGTAGGGTGCAAGCGTTAATCGGAATTACTGGGCGTAAAGCGTGCGCAGGCGGTTCGGAAAGAAAGATGTGAAATCCCAGAGCTTAACTTTGGAACTGCATTTTTAACTACCGAGCTAGAGTGTGTCAGAGGGAGGTGGAATTCCGCGTGTAGCAGTGAAATGCGTAGATATGCGGAGGAACACCGATGGCGAAGGCAGCCTCCTGGGATAACACTGACGCTCATGCACGAAAGCGTGGGGAGCAAACAGG) (Fig. S11C). Sequences classified as *Achromobacter* were computationally removed from the comparison data set. Alpha and beta diversity metrics were calculated as described below; however, samples were rarefied to 15,000 reads per sample.

Alpha diversity metrics were not significantly different between the contaminated samples amplified to 27 cycles and then computationally decontaminated of *Achromobacter* and the same re-extracted samples amplified to 30 cycles (Fig. S11D). Bray-Curtis and Jaccard beta diversity metrics also showed that computationally decontaminating samples had no impact on microbial community composition (Fig. S11E). Therefore, we proceeded to re-amplify contaminated DNA extraction plates 14–18 to 27 cycles and computationally remove *Achromobacter* sequences from the entire data set, as described below.

### Data processing and analysis

Raw sequence data generated as a part of this study were processed and analyzed using QIIME2 bioinformatics platform versions 2023.5 and 2024.10 (48). A total of 1,912 samples were sequenced, which included 50 negative extraction controls and 44 positive extraction controls. Raw data from each sequencing run (*n* = 8) were individually demultiplexed and quality filtered using the QIIME2 DADA2 plugin (49) with default parameters. All forward reads were truncated to 200 base pairs, while all reverse reads were truncated to 100 base pairs prior to merging. The frequency tables and sequence tables from each run were then merged. After denoising and quality filtering, a total of 56,621,198 reads remained, with an average of 29,613 reads per sample. Taxonomy was assigned using the QIIME2 feature-classifier classify-sklearn method (50) with a naïve Bayes taxonomy classifier trained on the V4 (515f/806rb) region of Greengenes2 backbone (v.2024.09) (51). ASVs classified as mitochondria, chloroplast, or *Achromobacter* were removed from the data set. The SEPP method from the QIIME2 fragment-insertion plugin was used to construct a rooted phylogenetic tree using the Greengenes2 reference phylogeny v.2022.10 (52).

Alpha and beta diversity metrics were generated using the QIIME2 phylogenetic-diversity plugin, using 5,000 reads per sample as the rarefaction depth. The lme4 v.1.1-38 (53) and lmerTest v.3.1-3 (54) linear mixed-effects models packages in R v.4.5.2 (55) were used to test for statistical differences in alpha and beta diversity. Alpha diversity metrics were evaluated using linear mixed-effects models to test the overall difference between indoor and outdoor microbial communities, using the formula “alpha diversity metric ~ decomposition environment + ADD + (1 | cadaver ID/body site).” The emmeans v.2.0.1 (56) R package was then used to calculate indoor and outdoor alpha diversity means adjusted for repeated measures and ADD. Differences in Bray-Curtis and unweighted UniFrac beta diversity were evaluated using the formula “PCoA axis 1 ~ decomposition_environment * ADD + (1 | cadaver ID/body site),” which also accounted for repeated cadaver measures and nested sample site (face and skin) measures. Microbiome Multivariable Associations with Linear Models 2 (MaAsLin 2) was used to identify differentially abundant taxa (57). ASV frequencies were collapsed to the species level (L7). MaAsLin 2 was run using the following parameters: fixed_effects = (“decomposition environment,” “ADD”) or (“previous maggot exposure,” “ADD”), random_effects = “Cadaver ID,” normalization = “TSS,” transform = “log.” Visualizations were created with JupyterLab v.4.1.5 using tidyverse v.2.0.0 (58), Plotly v.5.20.0, Seaborn v.0.11.2 (59), or Matplotlib v.3.6.0 (60) with Python v.3.8.16.

### Outdoor decomposition data set

Decomposition data generated during a previous outdoor study (10) was used here as a training data set to create a machine learning model for estimating time since death. Sequencing data were obtained through the open-source microbial study management platform Qiita (https://qiita.ucsd.edu/). Raw forward and reverse 16S rRNA gene amplicon sequences were obtained from Qiita study ID 14989. However, samples from Qiita prep ID 14929 were re-sequenced as described above and were included here (Qiita prep IDs 17684 and 17685). Sequences were processed as described above, except for truncation length. All forward and reverse reads were truncated to 150 bp prior to merging using DADA2.

### Blow fly organ data set

Blow flies were originally collected, dissected, and the organ-associated microbial communities were sequenced as part of a previous study that took place at Sam Houston State University (31). Raw 16S rRNA gene amplicon sequencing data generated as part of Deel et al. (31) were obtained from Qiita study ID 13301 (ENA project ID PRJEB61671). Sequences were quality filtered and merged, and taxonomy was assigned as described above.

### Microbial source tracking

SourceTracker2 (61) (https://github.com/caporaso-lab/sourcetracker2/pull/122) was used to estimate the proportion of microbial community contribution from fly organs to human cadavers placed during this study over the 21 days of decomposition. Cadaver skin and fly organ frequency tables were first rarefied to 5,000 reads per sample. Cadaver skin samples were grouped by decomposition environment (indoor vs outdoor), and then ASVs were averaged across each day of decomposition. Fly organ ASVs were averaged across each fly organ (labellum, tarsi, and oocyte). Fly organs were used as microbial sources, and the cadaver skin samples grouped by environment and day were used as sinks. The ST2 Gibbs function was run using default parameters. Mixing proportions were plotted using Matplotlib (v.3.6.0). Per-sink-feature-assignment tables were merged, and ASV counts were summed across each fly organ. Assignment counts were then plotted across days of decomposition for the 10 ASVs with the most counts. Plots for each fly organ and decomposition environment were generated using Plotly (v.5.20.0).

### Random forest regression modeling

An RFR model was trained on the Burcham et al. (10) decomposition data set to assess whether a model based only on outdoor skin samples can accurately predict the PMI of cadavers decomposing inside SHEDs. This model was then used to predict the ADD of indoor and outdoor skin samples collected as a part of this study (SHEDs data set). The Burcham et al. (10) frequency table was filtered to only contain skin samples (skin of face and hip), rarefied to 5,000 reads per sample, and ASVs were collapsed on taxonomy level 7 (species), which has previously been shown to provide the best performance (10). Skin samples from the face and hip of cadavers decomposing indoors and the paired outdoor control cadavers were used as the test set. The frequency table was also rarefied to a depth of 5,000, and ASVs were collapsed to the species level to match the training set. The Burcham et al. (10) table and the table from this study were then filtered to only contain features that were present in both data sets (*n* = 2,153). Random forest models were built using the scikit-learn package (v.0.24.1) (62). The best hyperparameters from Burcham et al. (10) (bootstrap=False, max_depth=None, max_features=0.2, n_estimators=1,000) were selected for training. This model was used to predict the ADD of the SHEDs skin data set. MAE was used to evaluate model accuracy by subtracting the predicted ADD from the actual ADD and taking the absolute value of all test set samples.

A cross-validation approach was used to evaluate the inclusion of indoor cadavers on model performance. Using LeaveOneGroupOut partitioning, we grouped the SHEDs skin data by cadaver so that samples from the same cadaver were not split between the train and test data sets. For each random forest regression model, one cadaver was withheld as a test set and a model was built using the remaining cadavers (*n* = 26). ADDs were then estimated for all skin samples in the test set using this 26-cadaver model. The test cadaver was then moved back into the training set, and a new cadaver was then assigned as the test set. This process was repeated until all cadavers (*n* = 27) were designated as the test set. All models were run using the hyperparameters described above. Feature importances were averaged across all 27 models. Categorical variables, such as decomposition environment, were label encoded to numerical values prior to training the models. Maggot ADD was calculated beginning from the first day of notable maggot presence as described above. General model accuracy was determined by averaging the mean absolute errors of all ADD predictions across all cadavers.

## Data Availability

All 16S rRNA gene amplicon sequencing data generated for this study is available through Qiita (https://qiita.ucsd.edu/) under study ID 13810 and through European Nucleotide Archive (ENA) project ID ERP172982. Raw amplicon data for the Burcham et al. (10) data set can also be found on Qiita under study ID 14929 or through ENA project accession number PRJEB62460. The fly organ data set (31) can be found through Qiita under study ID 13301 or ENA project accession number PRJEB61671. Greengenes2 data files are freely available on the Greengenes 2 server (https://ftp.microbio.me/greengenes_release/). Code used for data processing and visualization, as well as intermediate files, is available through Open Science Framework under project “Microbiome of Indoor vs. Outdoor Human Decomposition” (https://osf.io/f9v8p/).

## SUPPLEMENTAL MATERIAL

File S1 msystems.00681-25-s0001.xlsx Linear mixed-effects model results comparing diversity metrics across decomposition environment.

File S2 msystems.00681-25-s0002.xlsx MaAsLin2 differentially abundant taxon results.

File S3 msystems.00681-25-s0003.xlsx ANOVA results comparing model performance.

Supplemental Figures msystems.00681-25-s0004.docx Figures S1-S11.

ASM does not own the copyrights to Supplemental Material that may be linked to, or accessed through, an article. The authors have granted ASM a non-exclusive, world-wide license to publish the Supplemental Material files. Please contact the corresponding author directly for reuse.
