# Supplementary material for: Insects shape the cadaver decomposition microbiome and postmortem interval estimation accuracy
Source: mSystems. 2026 Apr 27;11(5):e00681-25. doi: 10.1128/msystems.00681-25 (PMC13185568; doi:10.1128/msystems.00681-25)
Supplement: Supplemental Figures — Figures S1-S11. [file msystems.00681-25-s0004.docx]

**Supplemental Figures**

**
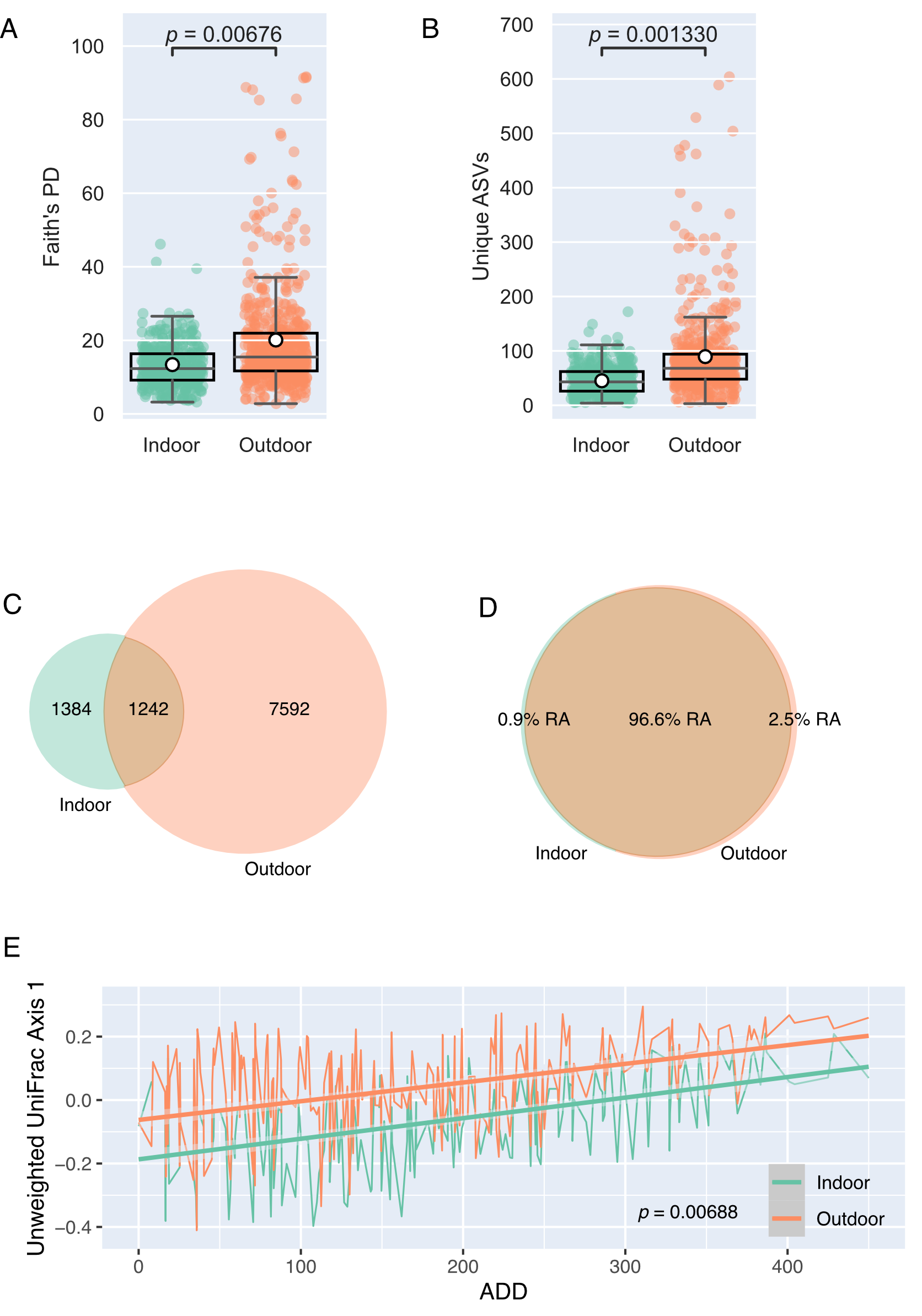
**

**Supplemental Figure S1. Alpha and beta diversity differences between decomposition environments. A)** Box plots of Faith’s phylogenetic diversity (PD) and **B**) number of unique ASVs comparing skin of indoor cadavers (teal) and outdoor cadavers (orange). Midline represents the group medians. White circle markers represent group mean values adjusted for cadaver ID and ADD. Linear mixed-effects models were used to test for significance between groups. **C)** Venn diagram shows the number of unique ASVs only found in indoor cadaver skin samples (*n* = 1384), outdoor samples (*n* = 7592), and across both decomposition environments (*n* = 1242). **D)** Venn diagram shows the unique indoor ASVs (*n* = 1384) only accounted for 0.9% relative abundance of indoor skin samples while the 7592 unique outdoor ASVs accounted for 2.5% relative abundance of outdoor samples. **E)** Mean Unweighted UniFrac PCoA Axis 1 coordinates are shown for indoor (teal) and outdoor (orange) cadaver skin samples across ADD with standard error (grey). A linear mixed-effects model was used to test for statistical significance between indoor and outdoor with cadaver ID used as a random intercept. Timepoints with multiple skin samples (face and hip) per cadaver were handled using nested random effects.

**
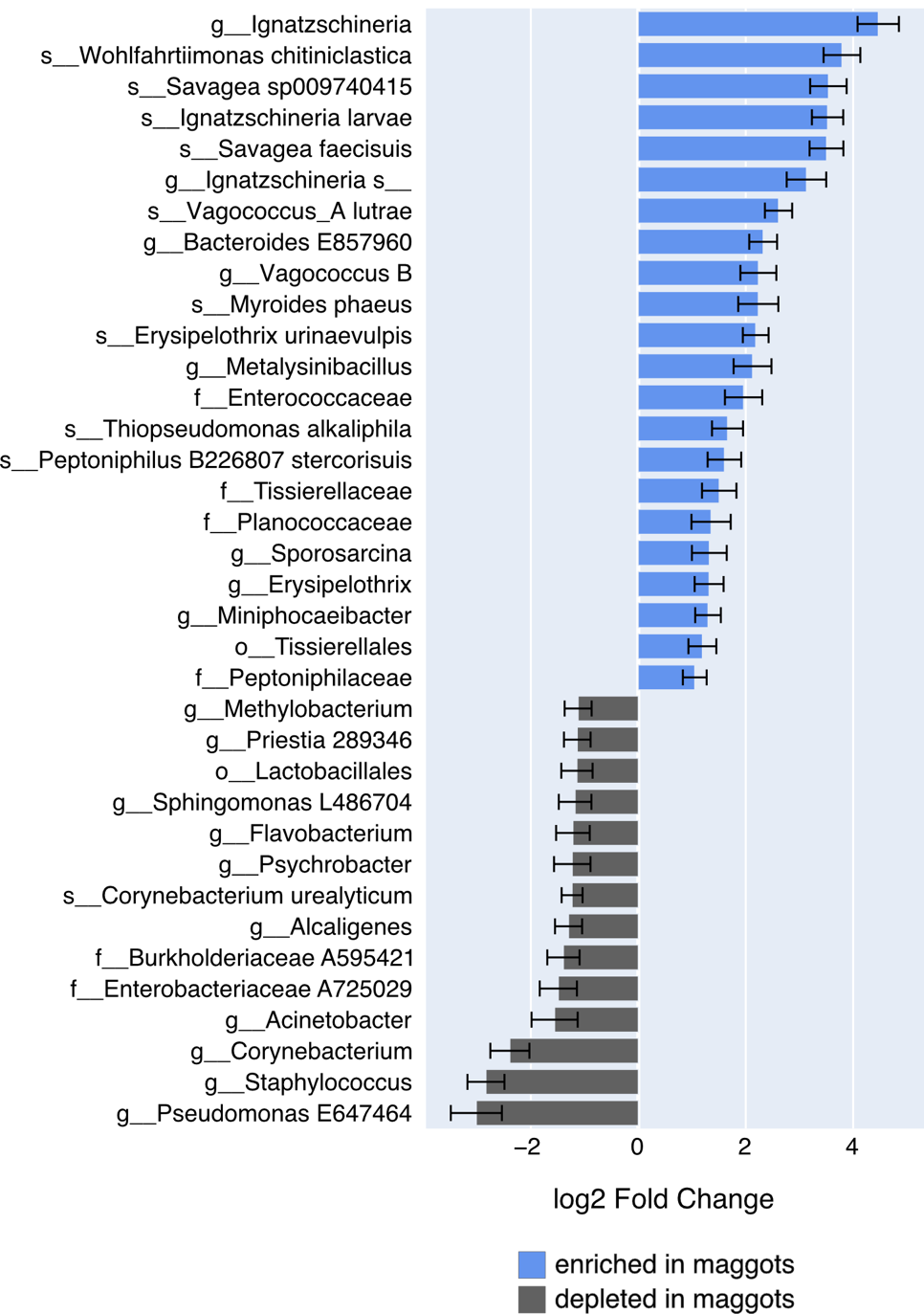
**

**Supplemental Figure S2. Taxa associated with maggot exposure.** Bar plot showing MaAsLin2 coefficient (log_2_ fold change) with standard error bars. ASVs were collapsed at species level and are shown at lowest taxonomic classification. Blue taxa were enriched in cadaver skin samples with previous maggot exposure while taxa in grey were enriched in maggot-free skin samples. Only taxa with >= +/- 1 log2 fold change and *q*-values < 0.05 are shown.


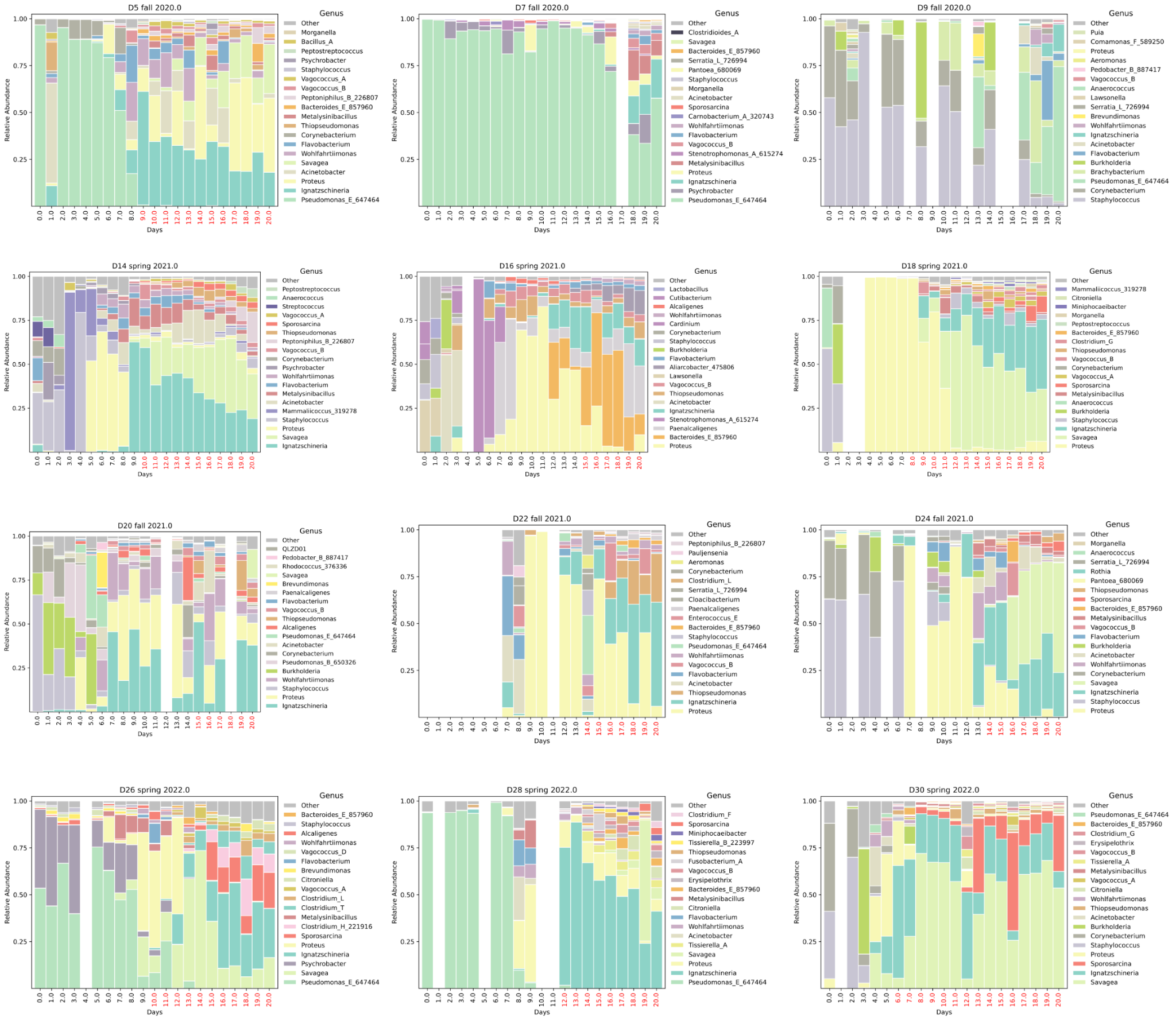


**Supplemental Figure S3. Indoor taxa bar plots.** Bar plots for each cadaver placed indoors are shown starting on the day of placement (day=0). Skin samples (face and hip) were averaged each day ASVs were grouped at the genus level. Samples were rarefied to 5,000 reads per sample. Blank bars indicate samples had fewer than 5,000 reads per sample at that time point. Days colored in red indicate the first day of notable maggot presence.

**
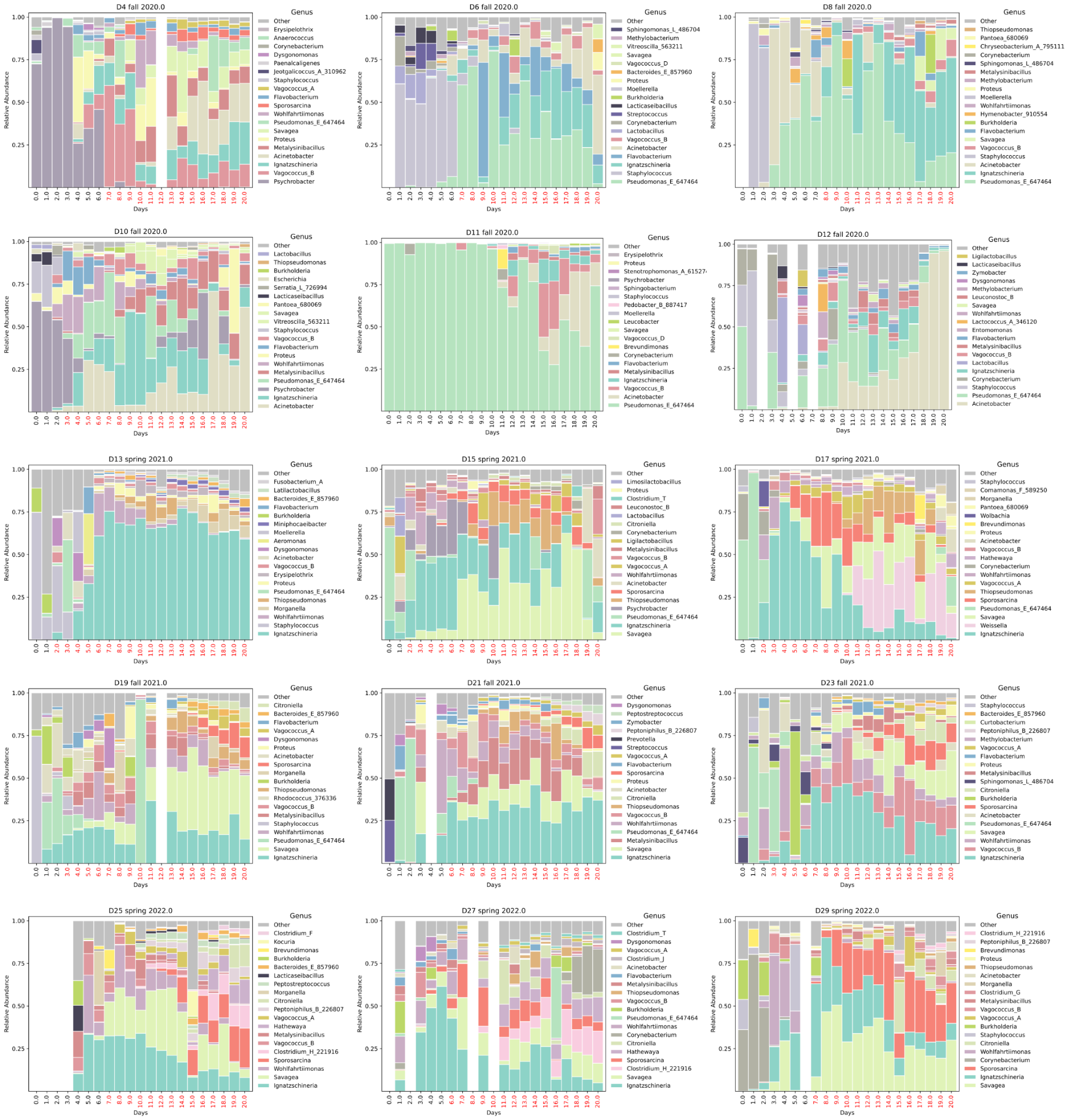
**

**Supplemental Figure S4. Outdoor taxa bar plots.** Bar plots for each cadaver placed outdoors are shown starting on the day of placement (day=0). Skin samples (face and hip) were averaged each day. ASVs were grouped at the genus level. Samples were rarefied to 5,000 reads per sample. Blank bars indicate samples had fewer than 5,000 reads per sample at that time point. Days colored in red indicate the first day of notable maggot presence.

**
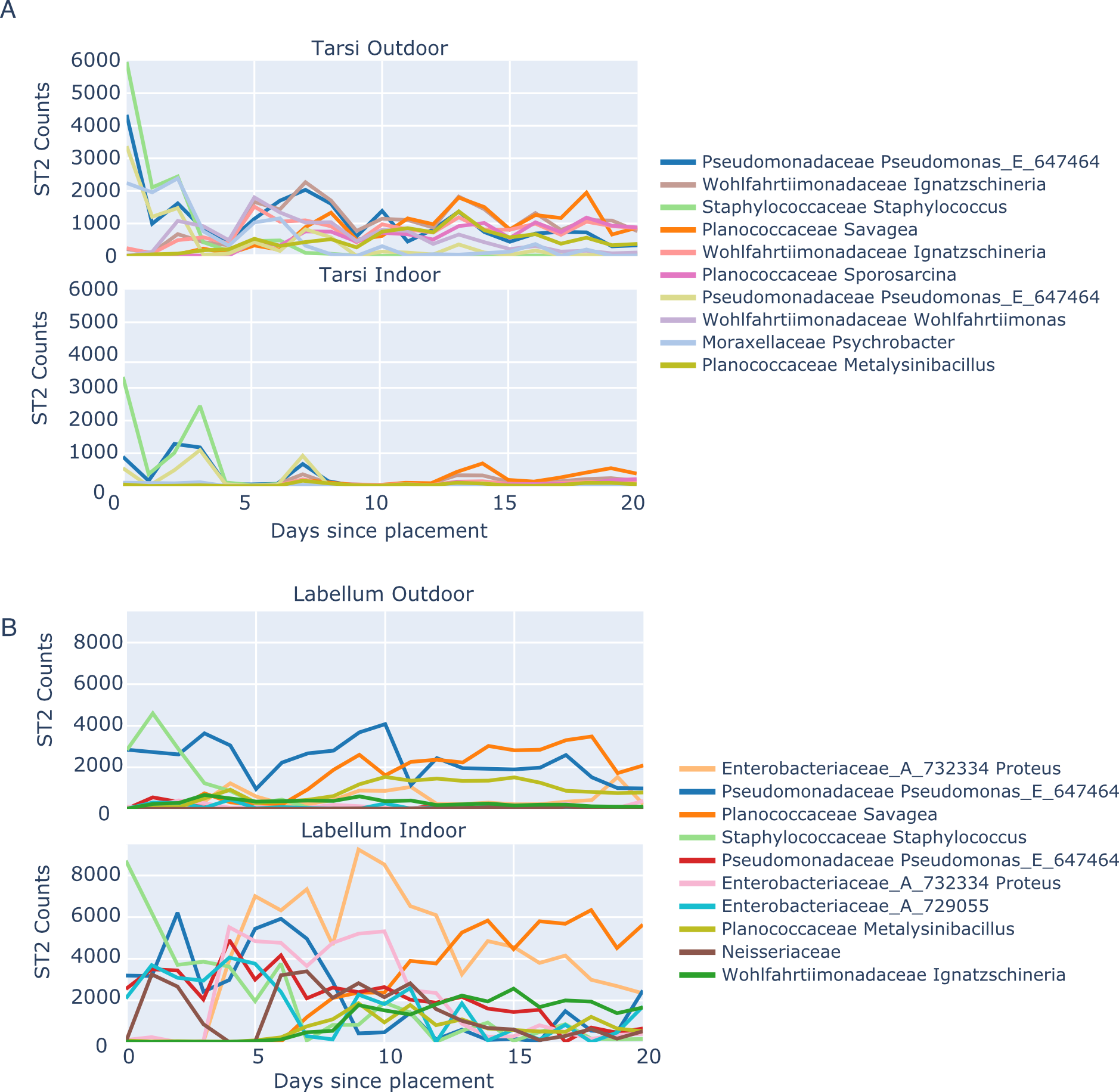
**

**Figure S5. ASV contributions to blow fly organ SourceTracker 2 (ST2) assignments.** Feature assignment counts (ST2 Counts) for the 10 ASVs with highest contribution assigned to tarsi (**A**) and labellum (**B**) are plotted across days of decomposition for outdoor (top) and indoor (bottom) cadaver skin. Taxa are labeled at their lowest taxonomic assignment—species if known, genus if unknown.

**
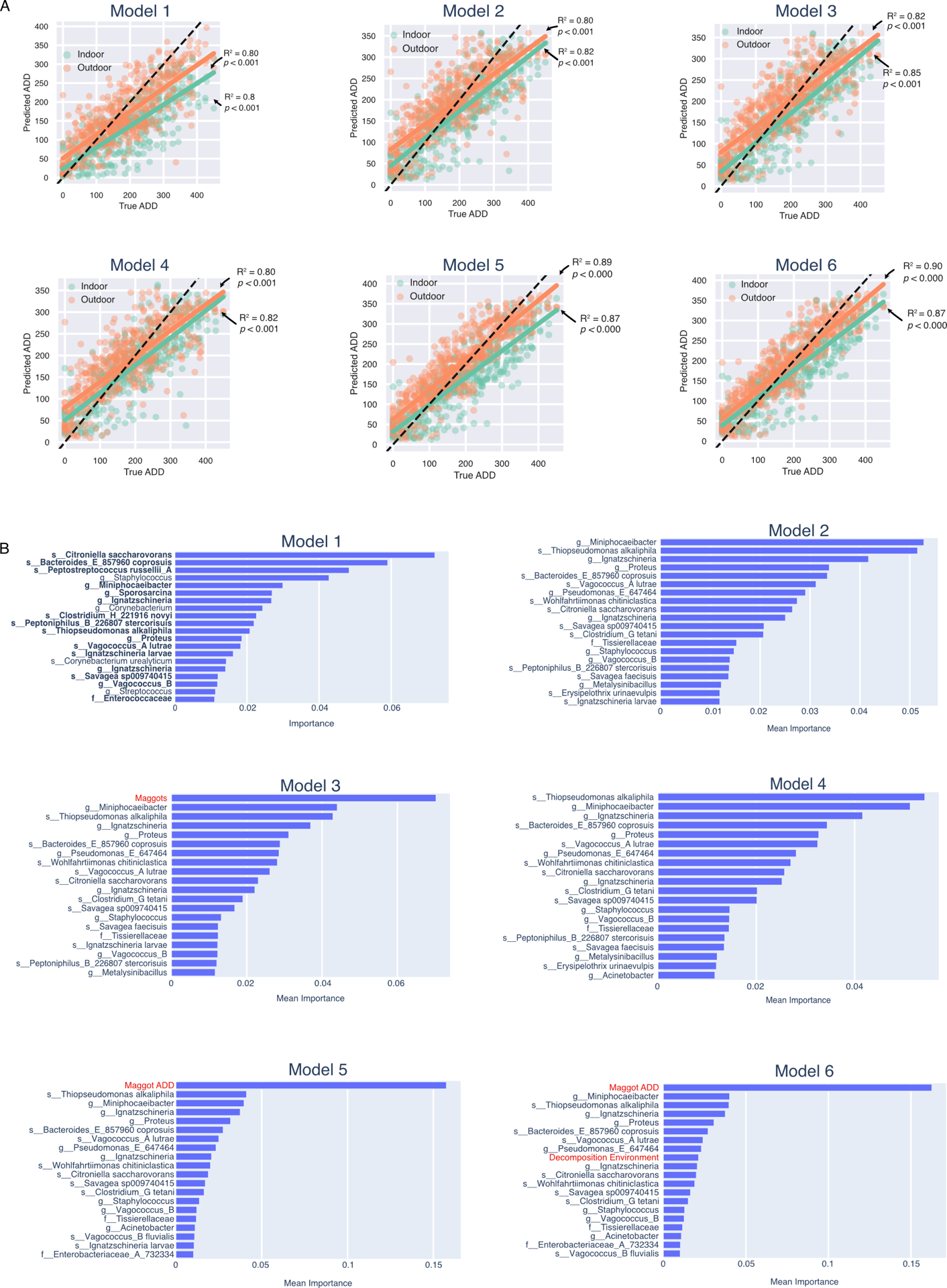
**

**Supplemental Figure S6. Important model features and performance. A**) Predicted ADDs are compared to true ADDs for indoor (teal) and outdoor (orange) skin samples across all models. Linear regressions for indoor and outdoor groups are shown. Dashed black line represents perfect prediction accuracy. **B)** The 20 most important features for each model are shown. Cross-validation was performed for models 2-6. Feature importances were averaged across all cross-validation folds (mean importance). Bolded taxa in **B Model 1** were also enriched in cadaver samples with notable maggot presence (see **Figure S2**). Features highlighted in red represent non-microbial, environmental features.

**
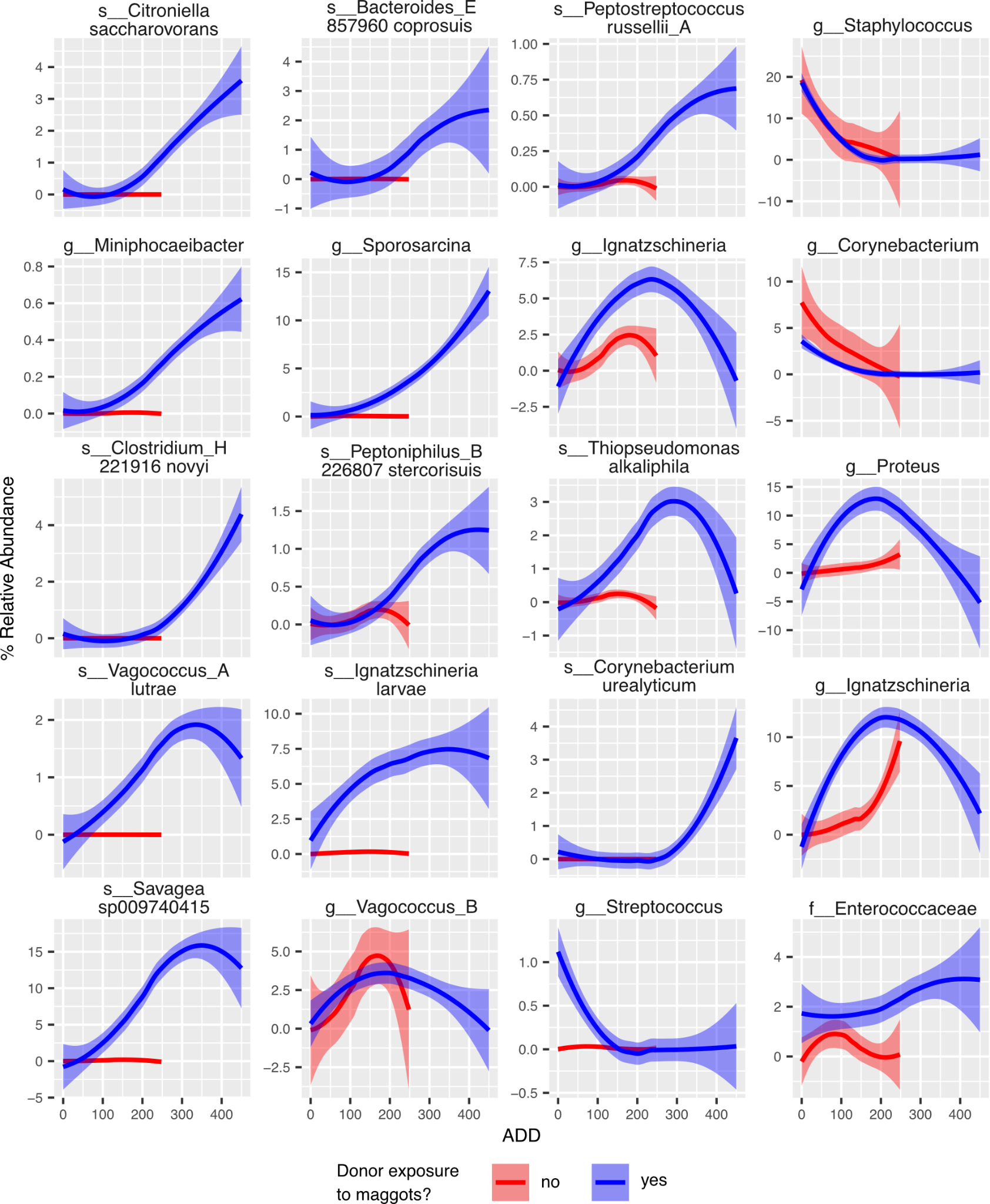
**

**Supplemental Figure S7. Several Model 1 important features are not present on cadavers that were never colonized by maggots.** The 20 most important Model 1 (Burcham et al. 2024) features are shown from most (top left) to least important (bottom right). The percent relative abundance of each feature is compared between the four cadavers (D7, D9, D11, and D12) with no notable maggot colonization (red) and cadavers with maggot mass (blue) across decomposition (ADD). Differences in total ADD (~250 vs ~450) are due to differences in weather—maggot-free cadavers (red) were placed in Fall 2020 during colder weather which resulted in lower total ADD (~250). Percent relative abundances were smoothed using LOESS regression.


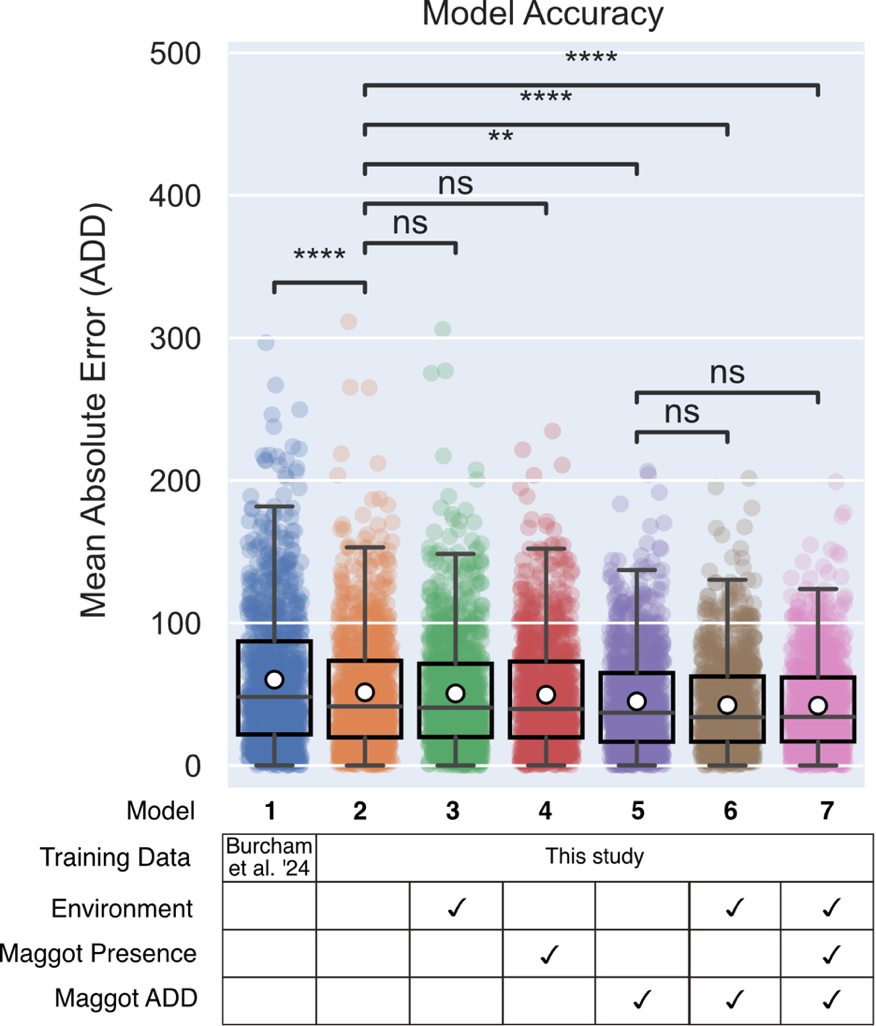


**Supplemental Figure S8. Overall model performance of combined indoor and outdoor cadavers.** Boxplots show mean absolute errors for each model that was constructed. White markers represent group means. Table summarizes which data types were included in each model. Training data column denotes which 16S rRNA amplicon dataset was used to train each PMI prediction model. In Model 1, 36 outdoor cadavers were used in training while 15 outdoor and 12 indoor cadavers were used in Models 2-7. Model 1 PMI prediction accuracy was evaluated by using all cadavers from this study as the test set. A leave-one-cadaver-out cross-validation approach was used to train and test Models 2-7. Decomposition environment, maggot exposure, and maggot ADD columns denote whether additional variables were included (“✓”) or excluded (“ “) from each model. ANOVA with post-hoc Tukey HSD correction for multiple comparisons was used to test for significance. *****p-*value < 0.0001, ****p*-value < 0.001 , **p*-value < 0.05; ns = not significant.

**
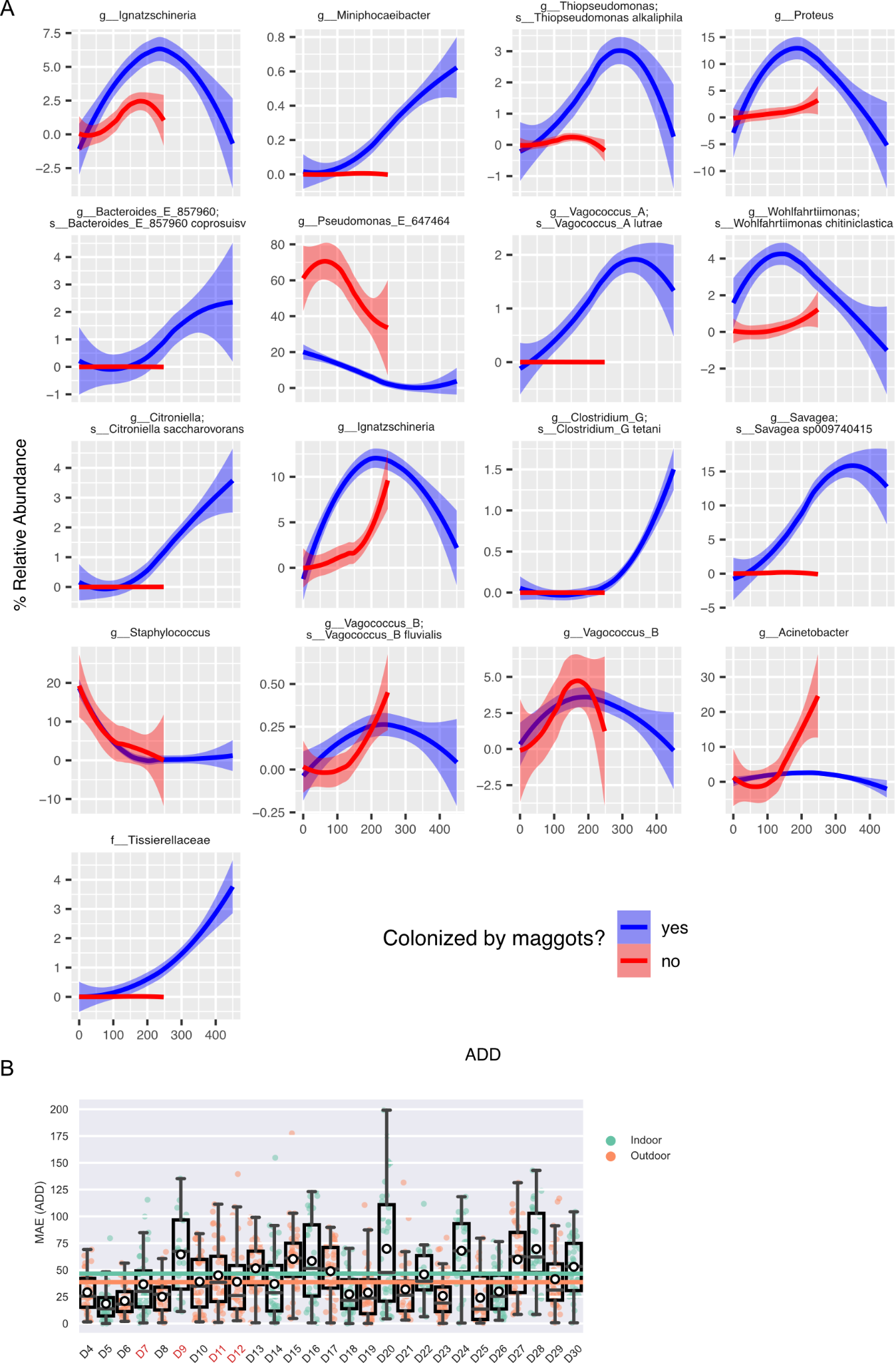
**

**Supplemental Figure S9. Several of the most important model features are also found in maggot-free cadavers.** The 17 most important microbial features from Model 7 (16S, environment, maggot presence, & maggot ADD) are shown from most (top left) to least important (bottom right). The percent relative abundance of each feature is compared between the four cadavers (D7, D9, D11, and D12) with no notable maggot colonization (red) and cadavers with maggot mass (blue) across decomposition (ADD). Differences in total ADD (~250 vs ~450) are due to differences in weather—maggot-free cadavers (red) were placed in Fall 2020 during colder weather which resulted in lower total ADD (~250). Percent relative abundances were smoothed using LOESS regression. **B)** Mean absolute error for each cadaver is shown. White marker reflects the mean value. Indoor cadavers are colored in teal and outdoor cadavers are colored in orange. Cadavers with no notable maggot colonization are labeled in red. Horizontal lines represent MAE of indoor (46.58 ADD) and outdoor cadavers (38.64 ADD).

**
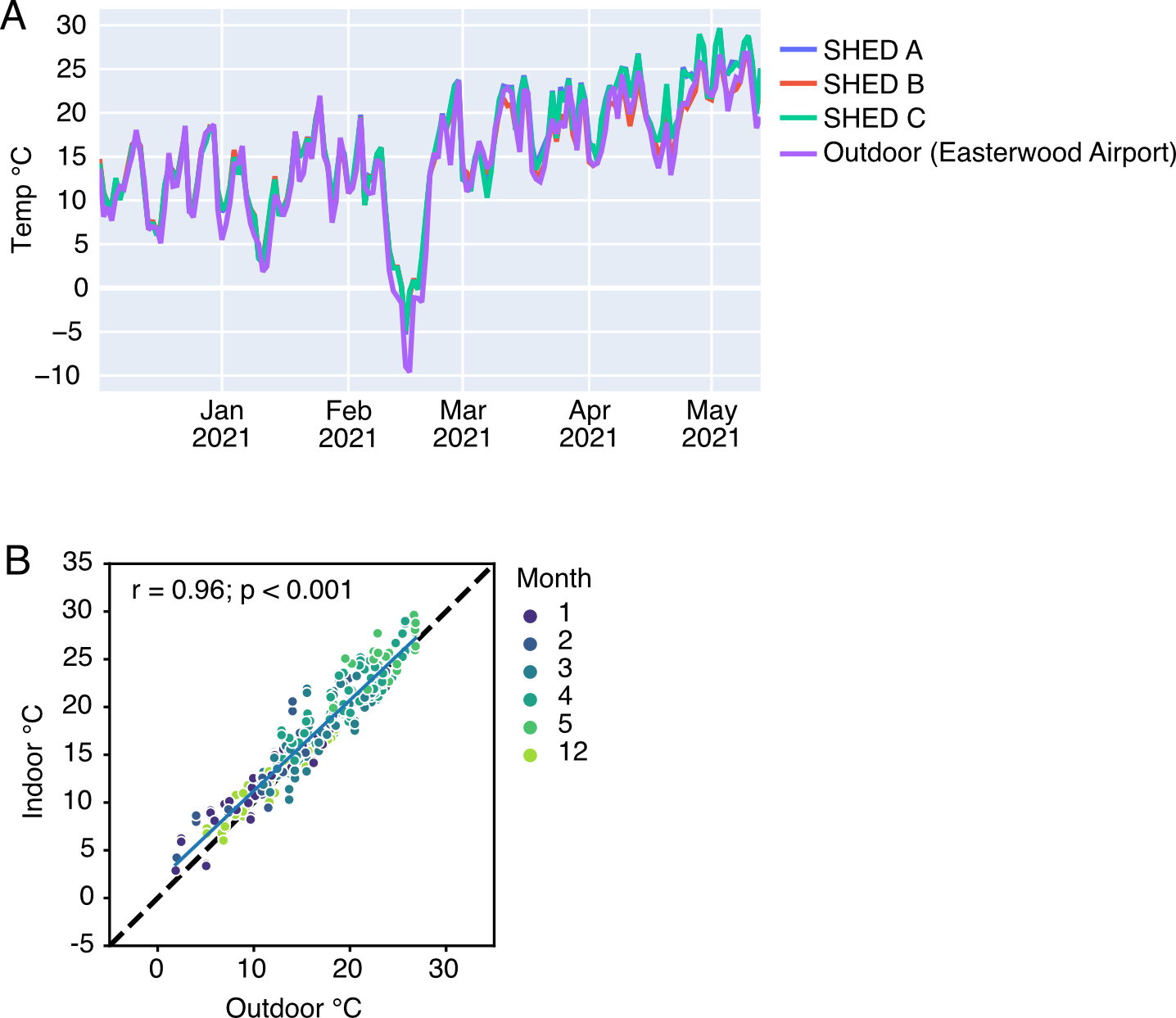
**
**Supplemental Figure S10. Indoor temperature assessment. A)** Average daily temperatures between December 02, 2020 and May 13, 2021. Temperatures were collected from inside each individual structure (SHED) and compared to outdoor temperatures. Outdoor temperatures were collected from Easterwood Airport located in College Station, Texas. **B)** Indoor temperatures were highly correlated with outdoor temperature. Pearson correlation was used to test for statistical significance. Solid blue line represents linear regression trend line between indoor and outdoor temperatures. Dashed line represents perfect correlation.


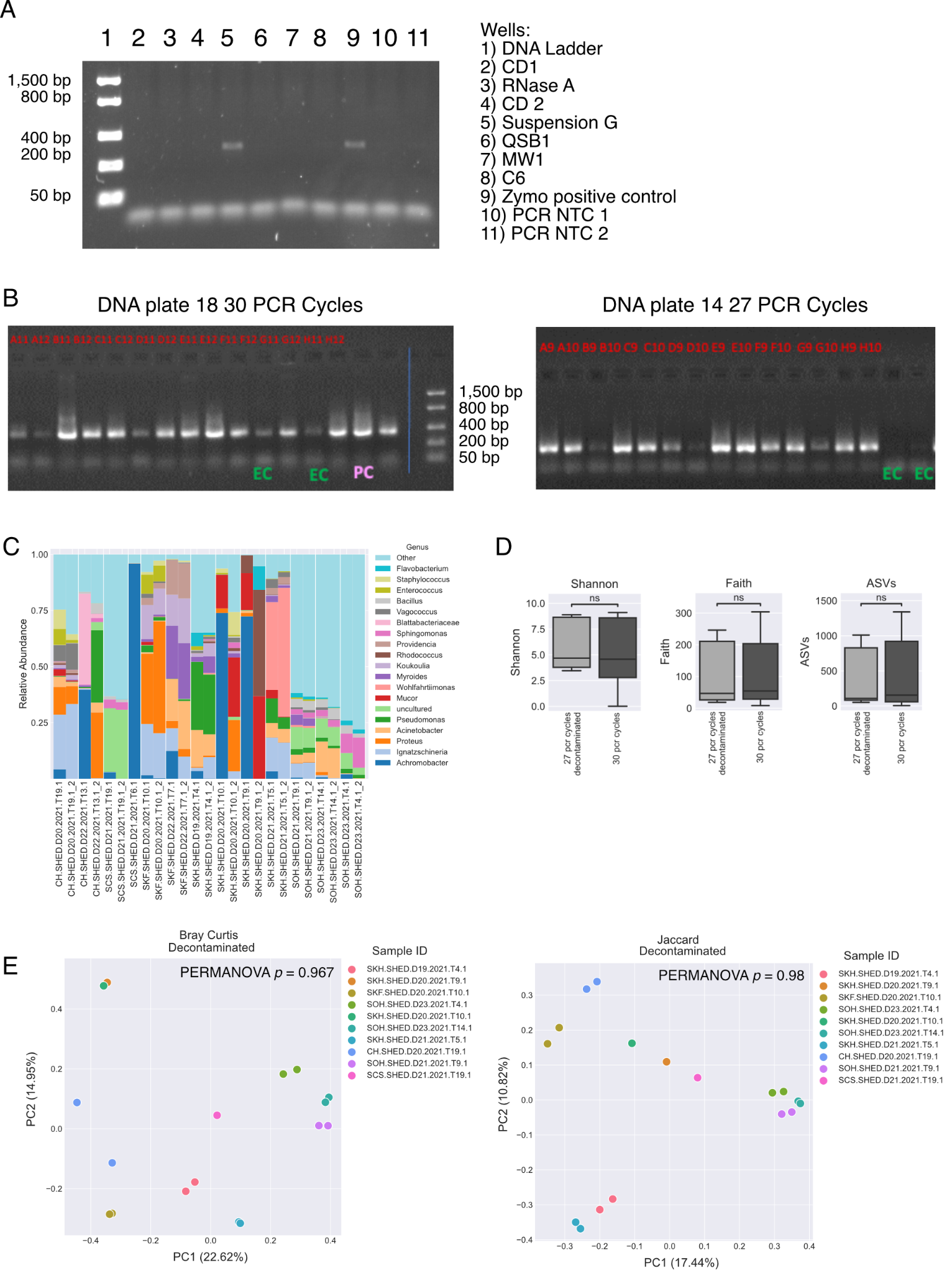


**Supplemental Figure S11. *Achromobacter* contamination mitigation. A)** Each reagent was tested for contamination (see methods). DNA agarose gel shows positive amplification in lane 5 from Qiagen MagAttract Suspension G beads. **B)** DNA agarose gel with amplicons from a subset of samples from extraction plate 18 is shown (left). Samples were amplified using 30 PCR cycles. Positive amplification was observed in the negative EC (extraction control) lanes. PC = positive control. Samples were re-amplified to 27 cycles. A subset of samples from DNA extraction plate 14 is shown on the right to demonstrate negative EC amplification was reduced following 27 cycles. **C)** Taxa bar plot shows *Achromobacter* sequences (dark blue) are absent from samples re-extracted with a clean DNA extraction kit (IDs ending in “_2”). **D)** *Achromobacter* was decontaminated from the samples amplified to 27 cycles and compared to the same samples re-extracted using a clean MagAttract PowerSoil Pro DNA isolation kit (Qiagen, cat no. 47109) amplified to 30 cycles. Shannon diversity (left), Faith’s phylogenetic diversity (center), and number of unique ASVs (right) were not statistically significant (ns) between groups following decontamination. Mann-Whitney test was used to test for significance between groups. **E)** PCoA plots showing Bray-Curtis (left) and Jaccard (right) beta diversity metrics showing distance between *Achromobacter* decontaminated samples amplified to 27 cycles and the same samples re-extracted with a clean DNA extraction kit amplified to 30 cycles. PERMANOVA was used to test for significance between indoor and outdoor skin samples.
